# Gap coupled symmetric split ring resonator based near zero index ENG metamaterial for gain improvement of monopole antenna

**DOI:** 10.1038/s41598-022-11029-7

**Published:** 2022-05-06

**Authors:** Md. Moniruzzaman, Mohammad Tariqul Islam, Md. Samsuzzaman, M. Salaheldeen M, Norsuzlin Mohd Sahar, Samir Salem Al-Bawri, Sami H. A. Almalki, Haitham Alsaif, Md. Shabiul Islam

**Affiliations:** 1 Department of Electrical, Electronic and Systems Engineering, Faculty of Engineering and Built Environment, Universiti Kebangsaan Malaysia, Bangi, Malaysia; 2 Department of Computer and Communication Engineering, Faculty of Computer Science and Engineering, Patuakhali Science and Technology University, Patuakhali, Bangladesh; 3 Department of Electrical Engineering, Faculty of Energy Engineering, Aswan University, Aswan, 81528 Egypt; 4 Space Science Centre, Institute of Climate Change, Universiti Kebangsaan Malaysia, 43600 Bangi, Selangor Malaysia; 5 Department of Electrical Engineering, College of Engineering, Taif University, P.O. Box 11099, Taif, 21944 Kingdom of Saudi Arabia; 6 Electrical Engineering Department, College of Engineering, University of Ha’il, Ha’il, 81481 Saudi Arabia; 7 Faculty of Engineering, Multimedia University (MMU), 63100 Cyberjaya, Selangor Malaysia

**Keywords:** Electrical and electronic engineering, Metamaterials

## Abstract

In this article, a symmetric split ring resonator (SRR) based metamaterial (MTM) is presented that exhibits three resonances of transmission coefficient (S_21_) covering S, C, and X-bands with epsilon negative (ENG) and near zero index properties. The proposed MTM is designed on an FR4 substrate with the copper resonator at one side formed with two square rings and one circular split ring. The two square rings are coupled together around the split gap of the outer ring, whereas two split semicircles are also coupled together near the split gaps. Thus, gap coupled symmetric SRR is formed, which helps to obtain resonances at 2.78 GHz, 7.7 GHz and 10.16 GHz with desired properties of the MTM unit cell. The MTM unit cell's symmetric nature helps reduce the mutual coupling effect among the array elements. Thus, different array of unit cells provides a similar response to the unit cell compared with numerical simulation performed in CST microwave studio and validated by measurement. The equivalent circuit is modelled for the proposed MTM unit cell in Advanced Design System (ADS) software, and circuit validation is accomplished by comparing S_21_ obtained in ADS with the same of CST. The effective medium ratio (EMR) of 10.7 indicates the compactness of the proposed MTM. A test antenna is designed to observe the effect of the MTM over it. Numerical analysis shows that the proposed MTM have an impact on the antenna when it is used as the superstrate and helps to increase the gain of the antenna by 95% with increased directivity. Thus, compact size, high EMR, negative permittivity, near zero permeability and refractive index makes this MTM suitable for S, C and X band applications, especially for antenna gain with directivity enhancement.

## Introduction

Metamaterial express exotic optical and electromagnetic properties when an interaction occurs with electromagnetic wave. Negative permeability, permittivity and refractive index are some exclusive properties of the metamaterial that can be used for various applications in communication systems of microwave frequencies. Antenna performance improvement^1–3^, radiation reduction^4,5^, design of absorber^6,7^, sensors^8,9^, high-frequency communications^10,11^, solar energy harvesting^12,13^, electromagnetic shielding^14^, metamaterial lenses^15^, etc. are some prominent applications of the metamaterial. Metamaterials can be classified as either single negative or double negative based on the fact that any one of permeability or permittivity is negative or both are negative. Artifical metamaterial can be constructed with metalic rings in association with splits. Geometrical structure of the MTM is an important parameter that governs resonance frequency along with the existance of negative permeability and permittivity.

Metamaterial (MTM) is extensively investigated by researchers to improve the performance of the antenna targeting various applications. In recent works, Sakli et al. has designed ultra-wideband antenna with MIMO configurations in which a complementary split-ring resonator is used to create isolation between antenna elements. By using this MTM, the transmission coefficient between two antennas decreases to − 43 dB, which is 1.87 times less compared to the same without using MTM. Moreover, the presented MIMO system with MTM helps to achieve size reduction and good diversity gain^16^. In another work, a metamaterial with near zero index is used to improve gain of a multiband antenna using MTM as a superstrate^17^. The metamaterial is also utilized by Das et al. to increase the bandwidth and gain of monopole antenna^18^, whereas Ke et al*.* have proposed a dielectric ring resonator(DRR) based quasi-Yagi antenna targeting broadband filtering applications in which good gain response is obtained by using wideband near-zero index characteristics of the metamaterial^19^. Moreover, in another article, an antenna is presented that involves metamaterial as superstrate for improving different parameters in antenna such as gain and bandwidth^11^. In this work, the metamaterial is used as radome to enhance the strength of the overall structure. Frequency can be reconfigured using diode switches in the metamaterial. In another article, the directivity of the antenna has been increased by using near zero permeability metamaterial^20^. Moreover, metamaterial helps to reduce the overall dimension of the antenna by about 11.4%. Moussa et al*.* proposes slotted metamaterial that helps to reduce mutual coupling between the antennas in MIMO system^21^. Moreover, metamaterial also helps to reduce the inter-element gaps. In another work, a metamaterial absorber is introduced with a four-element circularly polarized (CP) antenna array to reduce the mutual coupling effect. In this design, double-sided MTM absorbers walls having slotted cross patches are vertically placed between the antenna array elements that exhibit about 8 dB mutual coupling reduction^22^. A metasurface comprising with periodic metamaterial suspended over the MIMO antenna array for boosting up the antenna gain si present by Luo et al. the system provides 3 dB gain enhancement with mutula coupling redcution using neutralization line decouling element plance on feeding line^23^. Mishra et al. persented fractal antenna loading with metamaterial in which MTM helps to improve the bandwidth^24^. Jabire et al. describes a monopole antenna in which metamaterial is loaded to improve the bandwidth with reduction of mutual coupling^25^. Thus, metamaterials play a vital role in controlling different performance parameters of the antenna. A number of metamaterial structures have also been explored in various literature intending the applications in various fields in the microwave region^28–30^. Computational metamaterials providing the feasibility of wave-based computations in analog form with the massive parallel operation have been explored by Nejad et al.^31^*.* Dalgac et al. presents a chiral metamaterial as a sensor constructed with square and circular shaped resonator and works in X band and exhibits high sensitivity and selectivity to determine the quality of car lubricant oil^32^. On the other hand, a millimeter-wave range sensor based on metamaterial is presented by Qureshi et al. to characterize cooking oils, which show high transmission coefficient shifting when operating at 30 GHz^33^. A dual-function metamaterial is presented by Lu et al. that can be applied for vibration isolation along with energy harvesting^34^. Coskuner et al. has presented an impedance matching network based on a metamaterial-based transmission line to improve energy harvesting system operating around 2.4 GHz and 5 GHz^35^. A fabricated antenna loaded with metamaterial is presented by Ashyap et al. The metamaterial helps to diminish frequency detuning influence and attenuate backward radiation^36^. A metamaterial based antenna is presented in Ref.^26^ where a transmission line having negative permittivity and a splitted ring resonator are incorporated. Image theory is exercised to obtain impedance and the measured result exhibits impedance bandwidth of 16.36% having a gain of 5.62 dBi with an average efficiency of 72.1%. On the other hand, Ameen et al. presented a circularly polarized (CP) antenna with V-shaped metasurface^27^. This CP antenna exhibits a good gain of 5.76 dBi and it can be utilized for small satellite applications.

This article presents a metamaterial that is axis-symmetric and split rings or parts of the rings coupled near the split gaps intending to obtain the S_21_ resonances at S, C, and X-bands. These three bands cover the frequency ranges 2–4 GHz, 4–8 GHz, and 8–12 GHz for S, C, and X band, respectively, for various applications such as Radar, satellite Multimedia communications, etc. Particularly, the ISM band (2.4–2.45 GHz) is used for unlicensed devices such as cordless phones, wireless electronic devices, blue tooth, Wi-Fi communications. Moreover, in some countries, direct home to satellite television utilizes frequency band 2.5 to 2.7 GHz. For X-band satellite communication, 7.9–8.4 GHz is used for uplink, and 7.25–7.75 is used for the downlink. On the other hand, 10 to 10.5 GHz is allocated by ITU for amateur radio operations, whereas 10.45 to 10.5 GHz for amateur satellite operations. In our present design, we focus on the fact that the MTM can help to increase the antenna gain of a particular frequency band. For this purpose, we tried to keep the resonances of MTM around the frequency as mentioned above bands so that proposed MTM, when implemented with the microwave devices, can boost up the performances of those.

The proposed MTM exhibits three resonances of S_21_ at frequencies of 2.78 GHz, 7.7 GHz, and 10.16 GHz with − 10 dB bandwidth in the frequency ranges 2.38–3.1 GHz, 6.6–8.33 GHz, and 9.5–11 GHz, respectively, which can effectively influence to enhance the performance of microwave devices of our targeted frequency bands. The specific properties of the proposed MTM are: (i) Structural design of unit cell is simple, (ii) coupling between two outer layers from both ends of the split gaps of the outmost ring, named as gap coupling aids to adjust resonance frequencies at the target bands maintaining negative permittivity with near zero permeability and refractive index, (iii) The obtained EMR value of 10.7 indicates its compactness and makes it suitable for small-sized devices, (iv) The unit cell is symmetric about the two perpendicular axes through the center of the structure. Due to symmetric structure, the mutual coupling effect is reduced that provides similar resonances for different arrays, (iv) it exhibits good antenna gain enhancement properties when used as a superstrate with the antenna. A test antenna is designed, and the performance of the MTM on this antenna is examined through numerical simulation that provides about 2.45 dBi gain increment with boosting of the directivity of the antenna when the MTM array is used as the superstrate. The antenna is essentially a rectangular patch with slots. Two ground plane slots with the rectangular patch establish the resonances, but the overall shape of the antenna in both top and bottom perspectives is new. Furthermore, two inverted L-shaped parasitic elements at the bottom side help to modify the resonance frequency, and bandwidth as its length significantly affects both. The proposed antenna shows a wide bandwidth with marginal gain. But, when the proposed metamaterial is used with the antenna, significant gain enhancement is achieved within a broad bandwidth, verified through the experiments.

## Design of metamaterial and equivalent circuit modeling

This section includes a discussion on the proposed MTM that consists of the substrate material properties, resonating patch structure, simulation set up, step by step design process of MTM with the change of transmission and reflection coefficient. Furthermore, since the MTM unit cell exhibits multiband resonances indicating that the unit cell acts as LC resonance circuit, the equivalent circuit is also modeled and designed with validation using ADS in this section.

### Design and simulation of the proposed MTM

**Figure 1:**
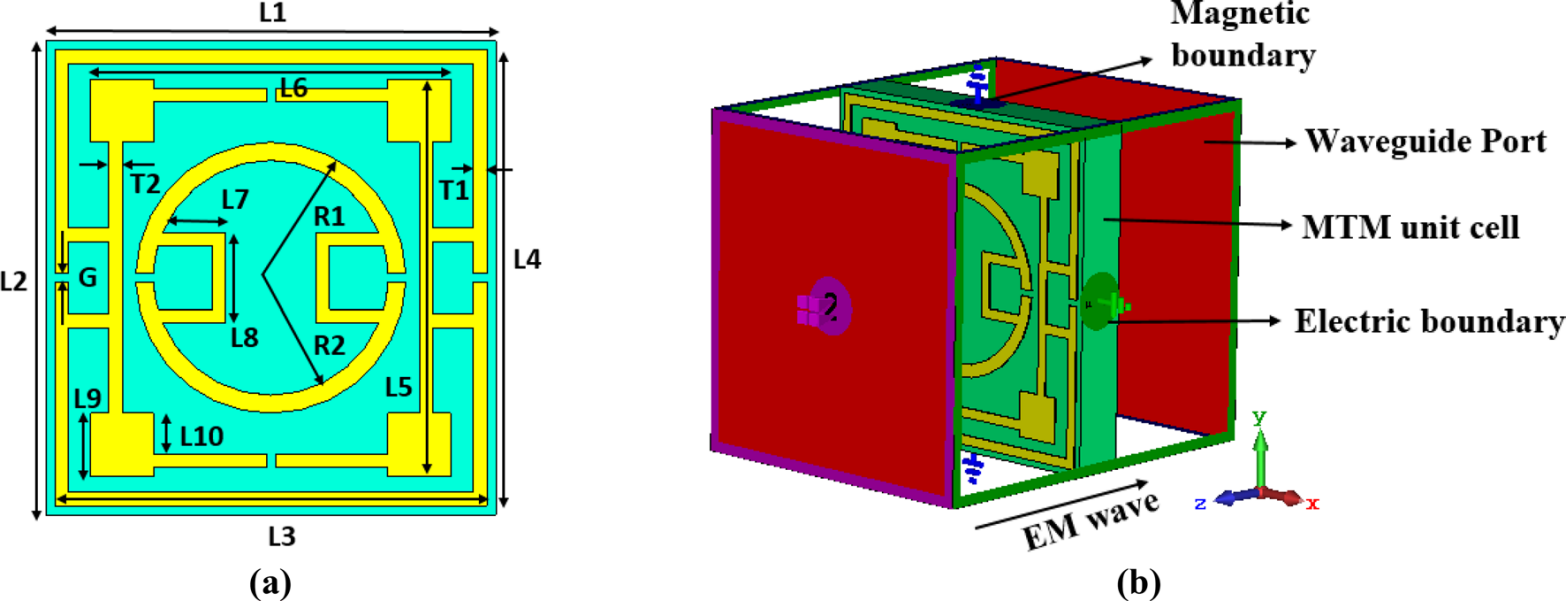
(**a**) Schematic layout of the unit cell and (**b**) simulation arrangement (CST STUDIO SUITE 2019, https://www.3ds.com/products-services/simulia/products/cst-studio-suite)^38^.

The proposed metamaterial(MTM) unit cell is designed on FR-4 substrate of 1.6 mm thick having a dielectric constant value of 4.3 with a loss tangent value of 0.025. The resonating patch is constructed at one side of the substrate using a copper metal having 0.035 mm thick. The MTM is originated on the substrate having a dimension of 10 × 10.5 mm^2^. As shown in Fig. 1a, the resonating patch is constructed with three split rings, the outer two are square-shaped, and the innermost one is circular. Two outer rectangular SRRs are coupled together by using metal strips at both ends of the split gaps of the outermost ring, as shown in Fig. 1a. Moreover, parallel inductive paths are created by coupling two ends of split gaps of the innermost circular ring. All these couplings contribute significantly to the resonances when electromagnetic waves are imposed on the MTM. Each SRR ring's dimension is selected, and the slip gaps and coupling metal strips are so positioned that the whole structure becomes axis-symmetric. The symmetric nature of the MTM helps to eliminate the harmonics and mutual coupling effect between the array elements when an array of the unit cells are employed for different uses. Table 1 presents the parameter values of different segments of the proposed MTM of Fig. 1a. The parameter values shown in Table 1 are finalized with numerous numerical simulations performed for the frequency ranges 2–12 GHz in CST microwave studio suite-2019 to obtained triple-band resonances of transmission coefficient (S_21_). The simulation arrangement in CST is presented in Fig. 1b, where two waveguide ports are used in Z-axis. In this arrangement, transverse electromagnetic (TEM) signal transmitted from one waveguide port incidents perpendicularly on the resonating patch. After transmitting through the MTM, the transmitted signal is received by another port. The electrical boundary is employed in X-axis in the simulation arrangement, on the other hand, the magnetic boundary is used in the Y-axis.

**Table 1:** Different segment dimensions and values.

Parameter	Size (mm)	Parameter	Size (mm)	Parameter	Size (mm)	Parameter	Size (mm)
L1	10	L2	10.5	L3	9.6	L4	10.1
L5	8.8	L6	8	L7	1.4	L8	2
L9	1.4	L10	0.9	T1	0.3	T2	0.3
G	0.2	R1	3	R2	2.6	–	–

The TEM waves represent a particular class of guided waves with neither E field component nor H filed component in the direction of propagation, meaning that E_z_ = H_z_ = 0 along the Z-axis. When this wave is incident upon the metamaterial, the response of the material depends on its properties such as permittivity, permeability. The time varying electric and magnetic flux densities in the material can be interrelated with the time varying electric and magnetic field using the mathmetical relations presented in Ref.^37^ which helps to understand the impact of the medium on some important parameters such as refractive index, wavenumber, and wave impedance. Electromagnetic field coupling occurs in a medium as time-varying electric charges are the sources of the magnetic fields, generating electric fields varied with time. These relations can be expressed through Maxwell’s equations presented in the differential form in Ref.^37^. The Maxwell’s equations indicate the coexistance of oscillating electric and magnetic fields, which initiates electromagnetic waves that travel through the medium. Now, when the incident wave is imposed upon the MTM, electromagnetic induction occurs and resonating phenomena is experienced as the split rings of it acts as resonantor. Thus the split ring resonator (SRR) can be demonstrated by an equivalent resonant circuit containg inductance, L and capacitance, C that offers resonances at some frequencies of SRR.

**Figure 2:**
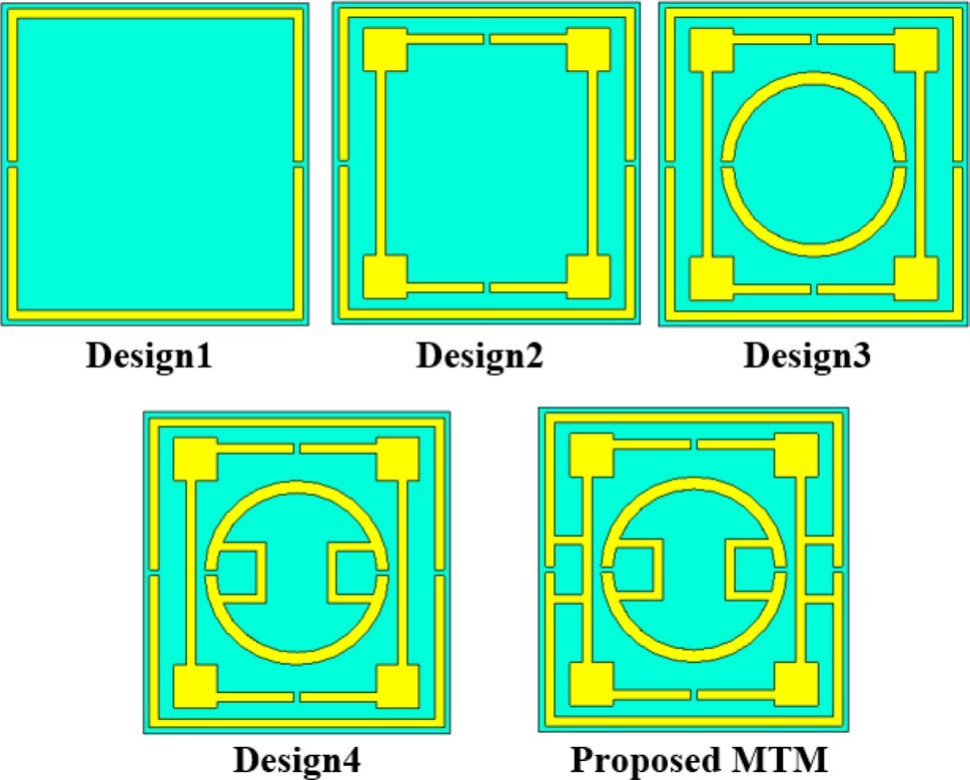
Evolution steps of the MTM towards proposed unit cell.

**Figure 3:**
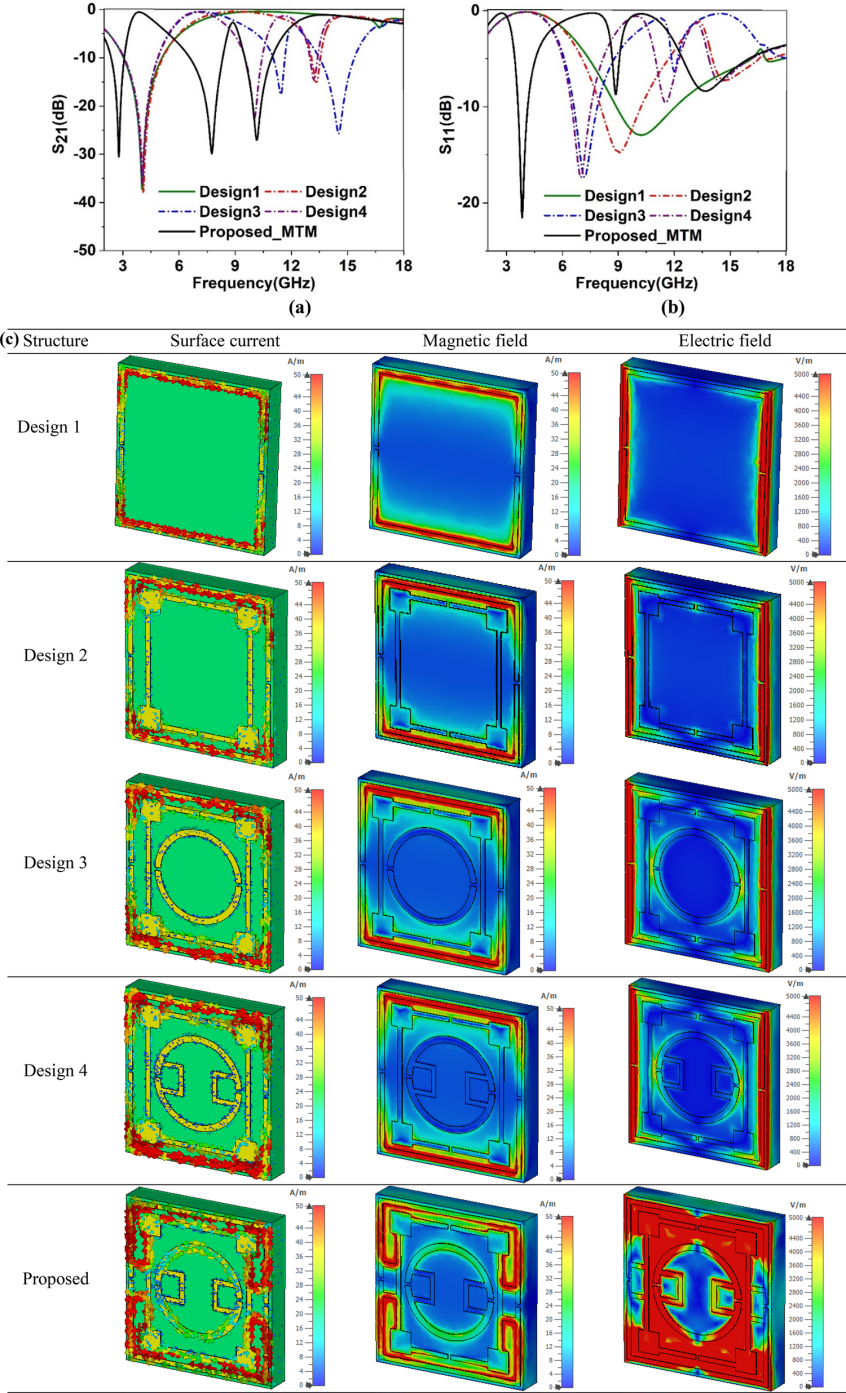
Scattering parameters for different design steps: (**a**) coefficiens of transmission (S_21_), (**b**) coefficients of reflection (S_11_). (**c**). Sufrace current, magnetic field and electric field distribution at 4.02 GHz for various design steps to the proposed MTM. (CST STUDIO SUITE 2019, https://www.3ds.com/products-services/simulia/products/cst-studio-suite)^38^.

The proposed MTM is designed through numerous numerical simulations with the modification of the length and width of different rings, and split gap distances since these parameters have an dominating impact on the resonance phenomena of the MTM. Moreover, inter ring distances and their interconnections are modified through trial and error basis to obtain the expected outcomes. Thus, the design finalization of the proposed MTM unit cell undergoes several design steps started with a square copper ring having two split gaps at vertical arms as displayed in design 1 of Fig. 2. This single ring causes a resonance of transmission coefficient (S_21_) taking place at 4.02 GHz, as shown in Fig. 3a. In design two, another square split ring is added that holds two splits at horizontal arms and square blocks at each corner, as illustrated in design 2 of Fig. 2. This insertion causes another addition resonance of S_21_ holding at 13.3 GHz. Moreover, owing to the mutual coupling between these two rings, primary resonance shifts slightly to 4.1 GHz. In the next step, one additional circular-shaped split ring is introduced (presented in design 3 of Fig. 2) that includes other resonances along with frequency shifts of the previous two resonances as consisted at 4.02 GHz, 11.43 GHz, and 14.5 GHz, as shown in Fig. 3a. In the succeeding step, two half circles of the innermost rings are coupled near the split gaps by using shunt copper strips as shown in design 4 of Fig. 2 with an effect of shifting of mid and high-frequency resonances at 10.02 GHz and 13.2 GHz as depicted in Fig. 3a. In the final step, two outermost rings are coupled adjoining the outmost ring’s gaps as illustrated in the proposed MTM of Fig. 2 with a formation of gap coupled symmetric split ring resonator MTM. This gap coupling causes shifting in the resonances at 2.78 GHz, 7.7 GHz and 10.16 GHz with an increasing effective medium ratio. Thus, the proposed MTM provides triple-band resonances within C, S, X-bands. The S_21_ response of the MTM for all design phases are also mentioned in Table 2, whereas reflection coefficients (S_11_) plots are presented in Fig. 3b for various design steps.

**Table 2:** Outcomes evaluation of proposed MTM for successive steps.

Substructure	Resonance frequency (GHz)	Bandwidth (GHz)	Resonance peak (dB)	Covering bands
Design 1	4.02	2.01	− 37.3	C
Design 2	4.1, 13.3	2.03, 0.45	− 38, − 15.4	C, Ku
Design 3	4.02, 11.43,14.5	1.8, 0.61, 1.86	− 36.1, − 17.63, − 25.7	C, Ku
Design 4	4.02, 10.04, 13.2	1.77, 0.89, 0.39	− 35.85, − 22.44, − 14.7	C, Ku
Proposed MTM	2.78, 7.7, 10.16,	0.7, 1.73, 1.45	− 30.5, − 29.86, − 27	S, C, X

The effect of different design steps is further analyzed through field analysis (magnetic and electric) and study of current at MTM at a particular frequency of 4.02 GHz, where resonance for design 1 occurs. As expressed in Fig. 3c shown in design 1, the horizontal sides of the outermost ring is susceptible to the magnetic field, and magnetic dipoles are created when electromagnetic waves are incident on the SRR, which in turn creates the current dipole with current flowing clockwise and anticlockwise through this two edges of outer ring. Electric field spreading is oriented in the vertical sides and forms an electric dipole. Therefore, magnetic and electric dipoles are linked to each other, and electromagnetic resonances of S_21_ occur at 4.02 GHz. In design 2, as extra ring is inserted inside earlier ring, this not only causes an additional resonance at 13.3 GHz but also modifies earlier resonance at 4.1 GHz. This is because of the mutual inductance and co-planar capacitance presented in Eqs. (7) and (8), respectively. This effect is evident from Fig. 3c, as it is noticed the opposite flowing currents through the inner ring compared to the outer ring. This opposition effect is dominant at four corners. Thus, this current causes a mutual inductance to the outer ring causing the modification of the total inductance and resonance frequency to 4.2 GHz. The electric field and magnetic field distribution for design 2 in Fig. 3c also exhibits the change compared to the same distribution of design 1. In design 3, an additional inner ring causes to reduce the mutual coupling effect on the outer ring; thus first resonance is restored at 4.02 GHz, whereas it has an impact on the second ring as shown electric and magnetic field distribution in design 3 of Fig. 3c. Design 4 has less impact on the field distribution at 4.02 GHz whereas, gap coupling in the proposed design modifies the current, electric field and magnetic field distribution drastically, and electric and magnetic dipole no longer exist in the outer ring and resonance shifts from this frequency to another frequency of 2.78 GHz.

### Equivalent circuit modeling of the proposed MTM

**Figure 4:**
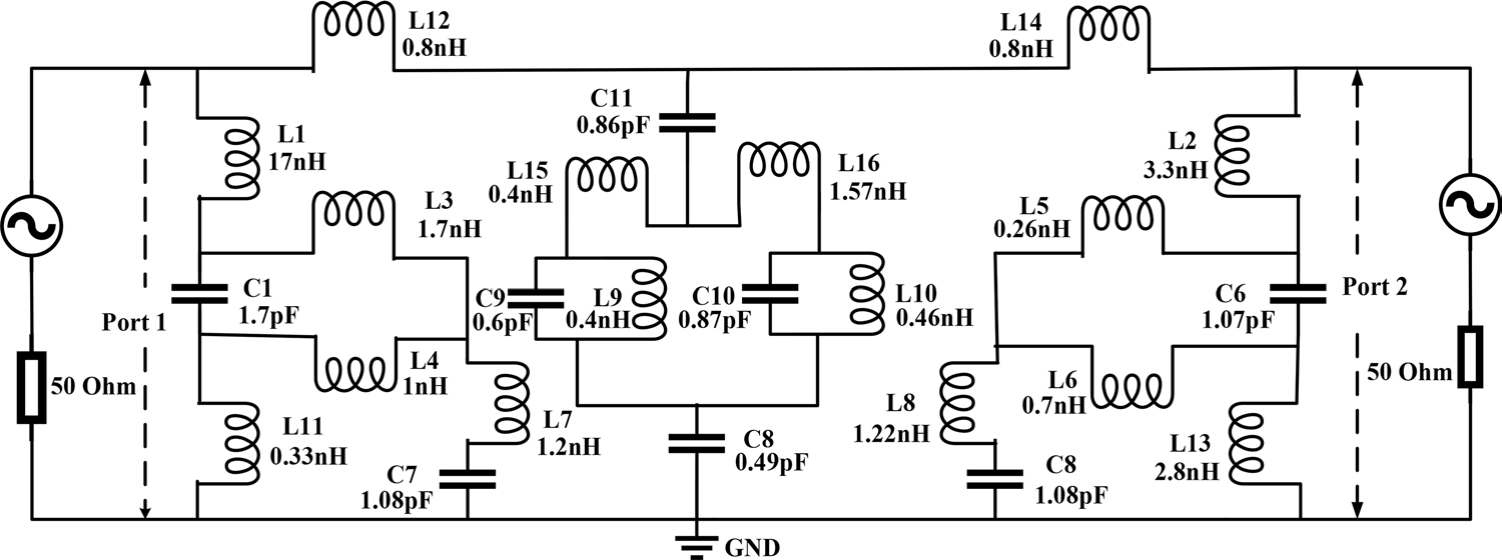
Equivalent circuit of MTM unit cell.

**Figure 5:**
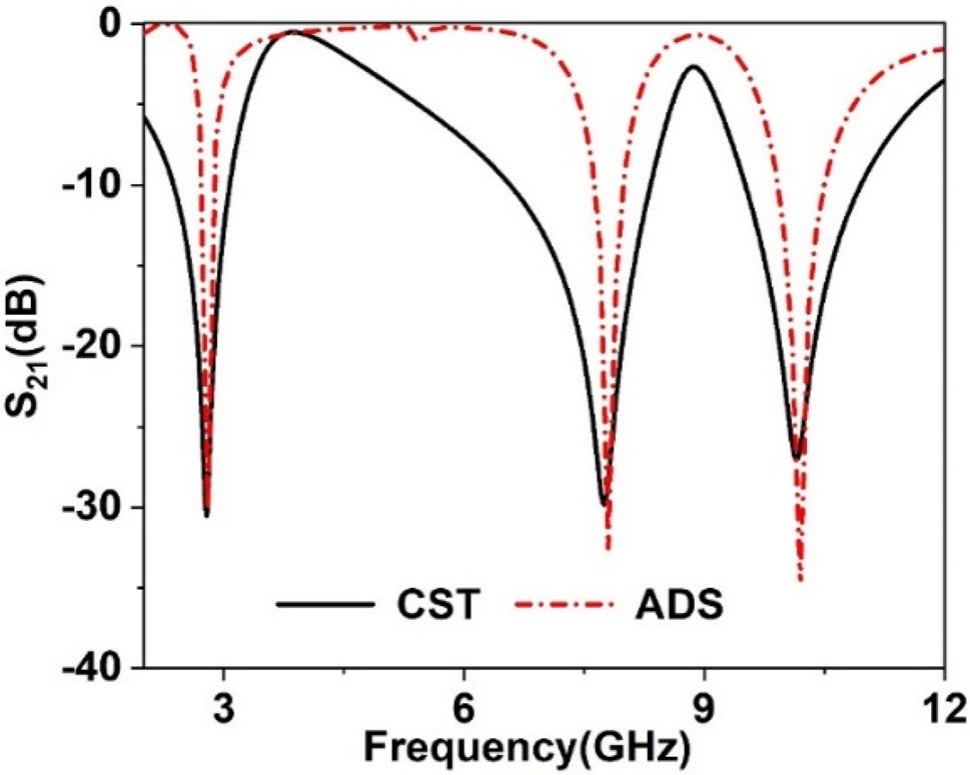
Validation of equivalent circuit through S_21_ comparison.

The equivalent circuit of the MTM can be drawn, considering it as analogous to LC resonance circuit^39^ as copper strips of the resonator exhibits inductive effect whereas split gaps have inductive effects. Due to these effects, various segments of the MTM exhibit different values of inductances and capacitances. Figure 4 shows the approximated equivalent circuit in which the inductances of different segments of the outer ring is represented by L1, L2, L11, L12, L13, L14, whereas C1 and C2 are the capacitances due to the split gaps of this ring. The effect of the middle ring is expressed by the L–C pairs L7 & C3 and L8 & C4, whereas L9, L10, L15, and L16 are the inductances and C5, C6 split gap capacitances for the innermost ring. L3, L4, L5, and L6 are the inductances due to the coupling of the two outermost rings. C7 and C8 are coupling capacitances between the innermost and outer rings. This circuit is then designed in Advanced Design Software (ADS) to check whether it resembles the proposed MTM structure or not by comparing the transmission coefficients (S_21_) obtained from the equivalent circuit and from the structure. Two ports are connected at the left and right sides of the equivalent circuit; those act as source and receiving ports. These transmitting and receiving ports are terminated with 50 Ω impedances. The simulation in ADS is started with the nominal values for the inductors and capacitors with 1 nH for each inductor and 1 pF for each capacitor. The component values are then adjusted using the tuning module in ADS as the tuning option provides the flexibility to enable the changing of one or more component values with a quick observation of the effect without emulating the circuit again. Thus, circuit parameter values are finalized through numerous tuning of the components when the obtained S_21_ exhibits resonance frequency closer to the S_21_ resonance in CST. A comparison of the S_21_ response obtained from the equivalent circuit with the same obtained in CST is shown in Fig. 5. As shown in Fig. 5, S_21_ of the equivalent circuit is well matched with the S_21_ of the MTM unit cell obtained in CST. As shown in Fig. 5, the bandwidth of resonances obtained from the equivalent circuit is narrower than the MTM response in CST. This is due to the fact that in the proposed MTM the inductance and capacitance are distributed all over the design domain. In contrast, in the equivalent circuit model, the components are considered lumped. Moreover, the resistive effects of the copper strips and inter-ring capacitances are ignored, which is distributive in nature.

## Result analysis of the proposed MTM

This section comprises various property analyses of the proposed MTM along with electromagnetic field and current distribution studies. Moreover, array performance is also studied as cluster of MTM cells arranged in a regualar pattern is utilized in many applications. Furthermore, the obtained result in measurement is also conferred, and an analysis is made on the presented MTM in comparison with other recent works on the MTM.

### Effective parameter analysis

The MTM property has been extracted by using CST post processing template that uses transmission (S_21_) and reflection coefficient (S_11_) coefficients to calculate permittivity, permeability and refractive index with the help of robust method where scattering parameters are concerned with by^40^:

1 $${S}_{11}=\frac{{R}_{01}\left(1-{e}^{i2n{k}_{0}d}\right)}{1-{{R}_{01}}^{2}{e}^{i2n{k}_{0}d}},$$

2 $${S}_{21}=\frac{\left(1-{{R}_{01}}^{2}\right){e}^{in{k}_{0}d}}{1-{{R}_{01}}^{2}{e}^{i2n{k}_{0}d}},$$

3 $${\mathrm{where}, \; R}_{01}= 1/{z+1},$$

4 $${e}^{in{k}_{0}d}=X\pm i\sqrt{1-{X}^{2}},$$

5 $$X=1/{2S}_{21}\left(1-{S}_{11}^{2}+{S}_{21}^{2}\right),$$

6 $$\mathrm{Impedance},z=\pm \sqrt{\frac{{\left(1+{S}_{11}\right)}^{2}-{{S}_{21}}^{2}}{{\left(1-{S}_{11}\right)}^{2}-{{S}_{21}}^{2}}},$$

7 $$\mathrm{Refractive \; index},n=\frac{1}{{k}_{0}d}{\left\{\left[{\left[ln\left({e}^{in{k}_{0}d}\right)\right]}^{{^{\prime}}{^{\prime}}}+2m\pi \right]-i{\left[ln\left({e}^{in{k}_{0}d}\right)\right]}^{^{\prime}}\right\}}$$

where (.)′ is real part, (.)″ is the imaginary part, m is an integer associated to real part of refractive index. Two other effective parameter permeability and permittivity can be calculated by using the equations:

8 $$\mathrm{Permeability}, \mu =nz,$$

9 $$\mathrm{Permittivity}, \; \varepsilon =n/z.$$

**Figure 6:**
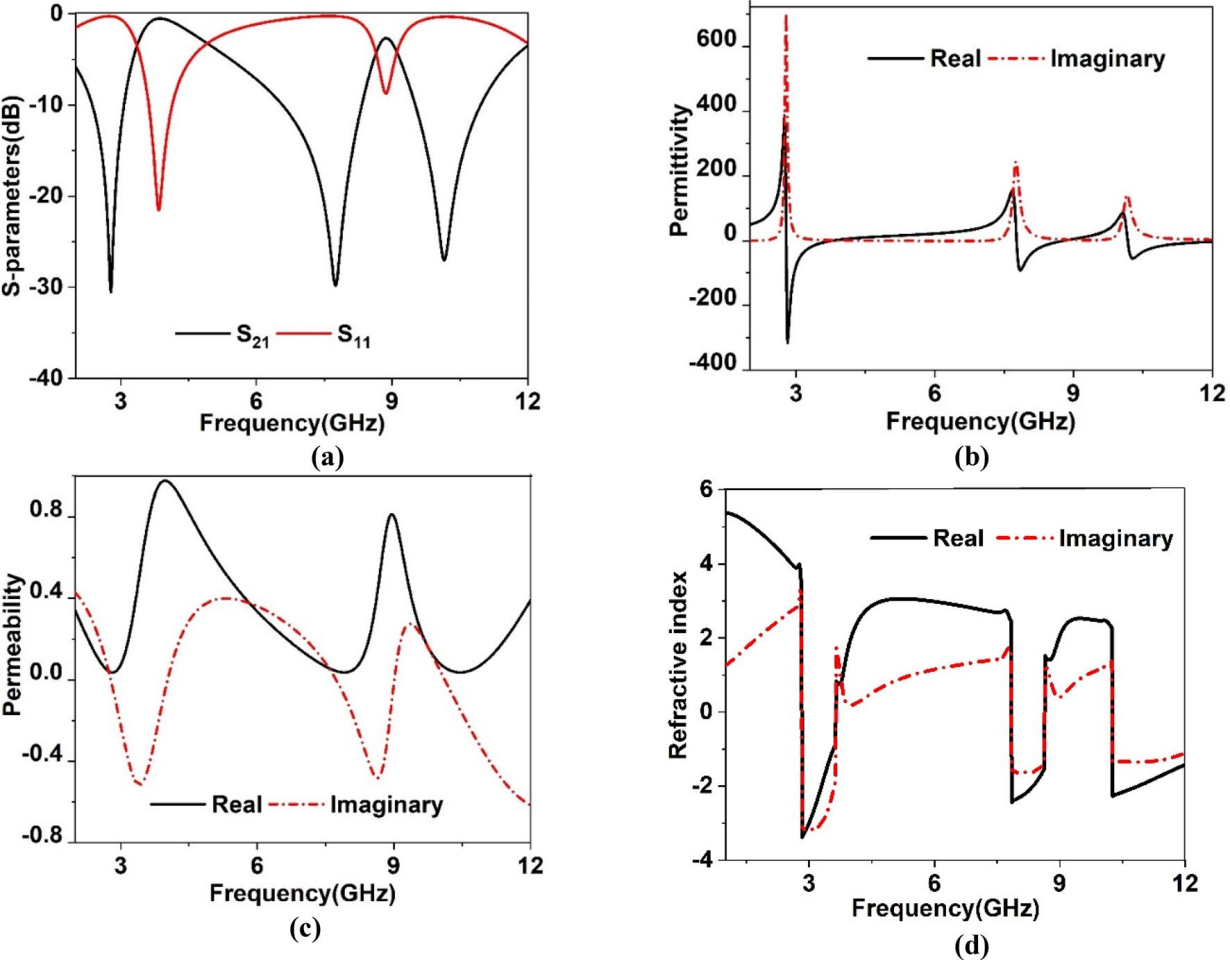
(**a**) Reflection and transmission coefficient plot (**b**) permittivity (**c**) permeability (**d**) refractive index.

By using Eqs. (7)–(9) the effective parameters are determined using post processing module in CST. The result obtained from the CST related to scattering parameters, permeability, permittivity and refractive index are depicted in Fig. 6a–d. As expressed in Fig. 6a, three resonances of transmission coefficient occur at 2.78 GHz, 7.7 GHz and 10.16 GHz, whereas S_11_ resonances occurred at 3.86 GHz and 8.85 GHz. It is witnessed that real component of the permittivity is negative within frequency ranges 2.78–3.75 GHz, 7.77–8.76 GHz and 10.17–12.74 GHz and obtained bandwidths are 0.97 GHz, 0.99 GHz and 12.57 GHz, respectively as expressed in Fig. 6b. Thus, comparatively wider bandwidth is obtained for negative values of permittivity for three different bands within S, C, X bands. The permeability plot depicted in Fig. 6c show near zero permeability with a magnitude less than 0.1 in the vicinity of 2.5–3.05 GHz, 7.27–8.3 GHz and 9.9–11.2 GHz which indicates wide bandwidth for near zero permeability. Minimum values of permeability are 0.04, 0.03 and 0.025 at 2.85 GHz, 7.94 GHz and 10.53 GHz, respectively. Additionally, as depicted in Fig. 6d, real component of the refractive index shows negative values within the negative permittivity frequency regions and near zero magnitudes is noticed in the vicinities of frequencies 2.82 GHz, 3.63 GHz, 7.84 GHz, 8.65 GHz, 10.25 GHz and 12.3 GHz. Thus, the proposed MTM reveals epsilon negative (ENG) behavior with both permeability and refractive index near zero, making it suitable for antenna applications for gain enhancement.

### Surface current, electric field and magnetic field analysis

**Figure 7:**
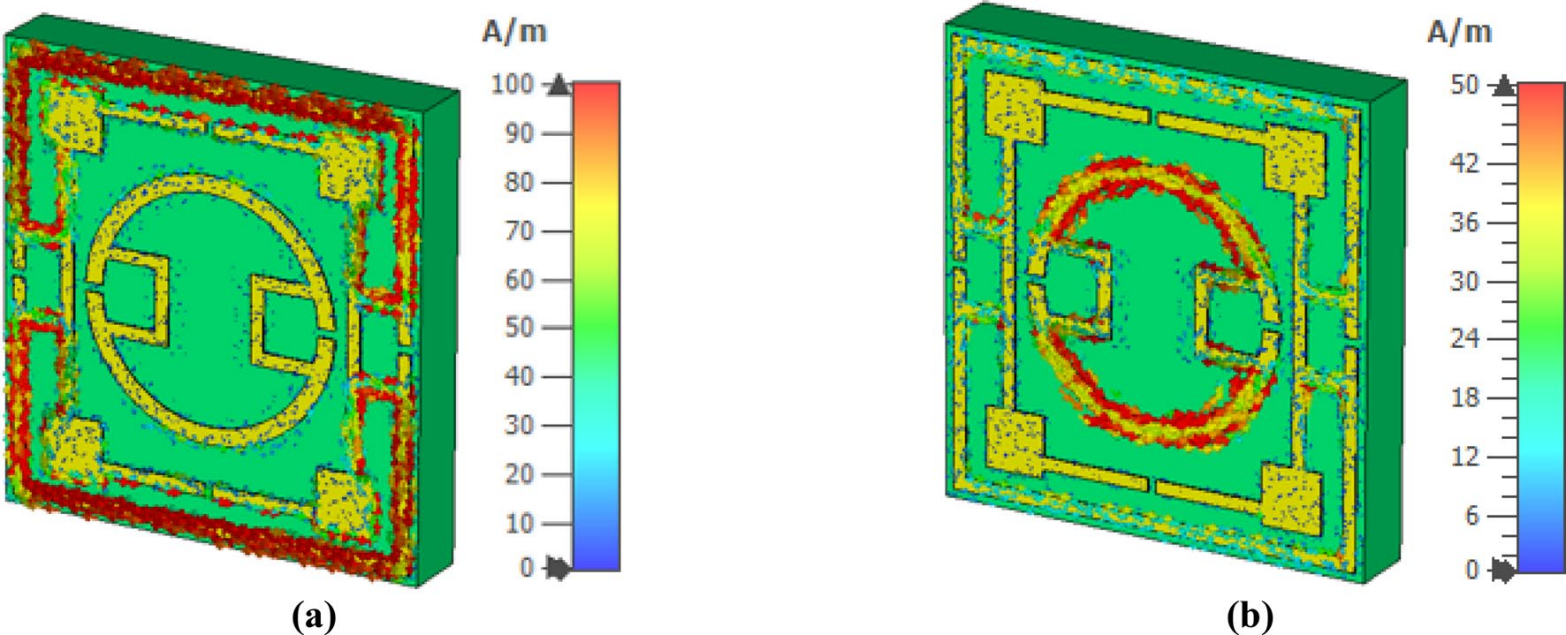
Surface current distribution for two different frequencies of S_21_ resonances: (**a**) 2.78 GHz, (**b**) 10.16 GHz (CST STUDIO SUITE 2019, https://www.3ds.com/products-services/simulia/products/cst-studio-suite)^38^.

**Figure 8:**
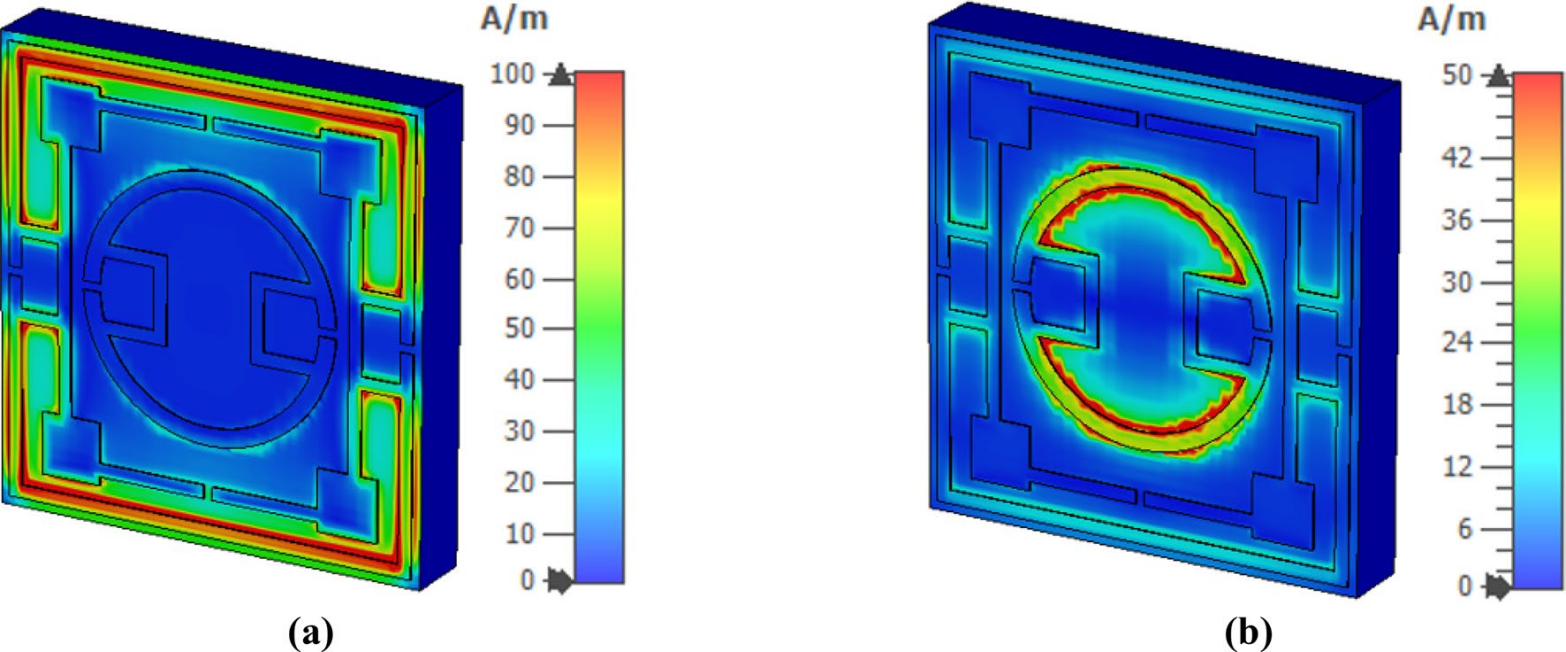
Magnetic field distribution for two different frequencies of S_21_ resonances: (**a**) 2.78 GHz and (**b**) 10.16 GHz (CST STUDIO SUITE 2019, https://www.3ds.com/products-services/simulia/products/cst-studio-suite)^38^.

**Figure 9:**
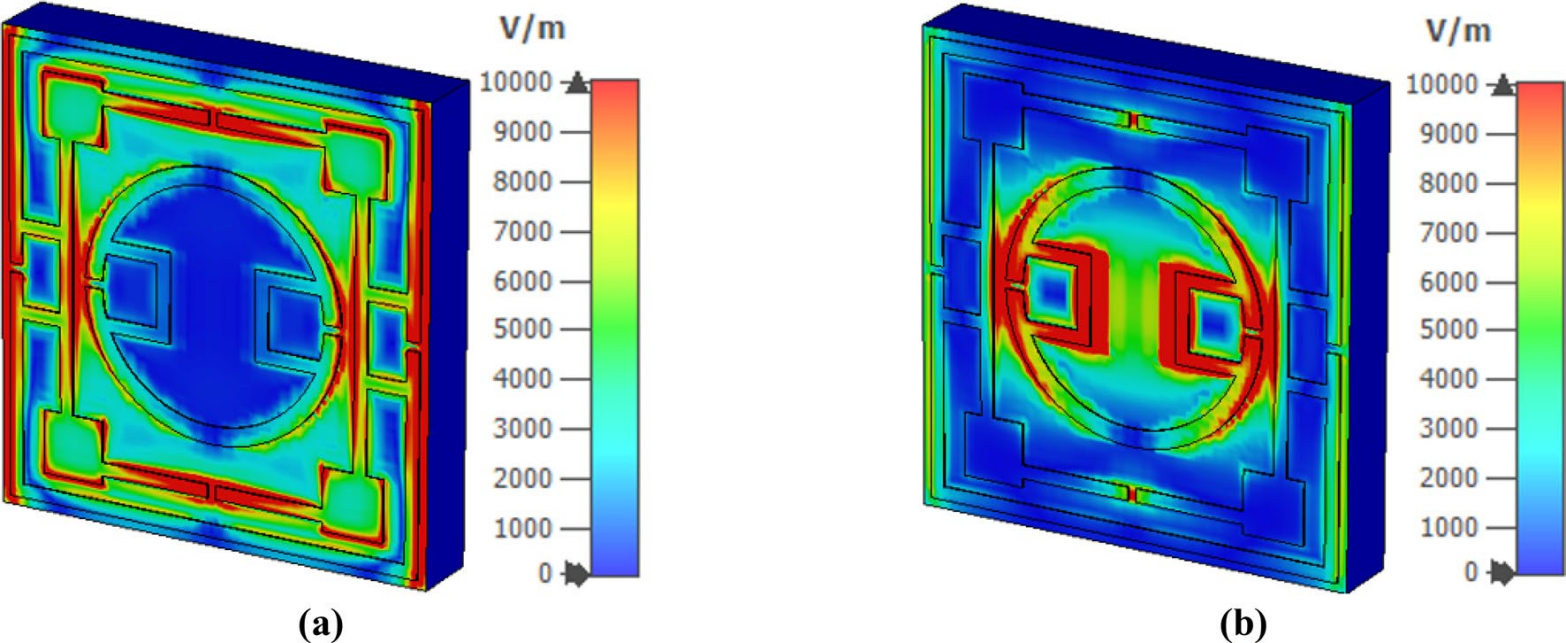
Electric field distribution for two different frequencies of S_21_ resonances: (**a**) 2.78 GHz and (**b**) 10.16 GHz (CST STUDIO SUITE 2019, https://www.3ds.com/products-services/simulia/products/cst-studio-suite)^38^.

The interaction of the electromagnetic wave with the metamaterial and its effects on resonances can be realized by analyzing various fields (electromagnetic) and current. Figure 7 shows the surface current spreading at 2.78 GHz and 10.16 GHz. In contrast, magnetic field and electric field distribution at these frequencies are presented in Figs. 8 and 9, respectively. The interrelation between various fields and currents can be explained with the help of Maxwell’s equations^37^. As shown in Fig. 7a, a strong surface current flows through the outermost ring at 2.78 GHz. The lower and upper halves of these ring currents form a circular current with the second ring though the current density in the second ring is low. The upper current loop forms circulating current in the anticlockwise direction, whereas the lower loop bears current in clockwise current. These currents induce a strong magnetic field surrounding the outer ring, as displayed in Fig. 8a. Likely, Fig. 9a shows the corresponding electric field distribution, indicating that a strong electric field exists at two vertical arms forming the electric dipoles triggering the resonance at 2.78 GHz. At this frequency, a strong magnetic field also exists at different portions of the middle ring and nearest portions of the middle and inner rings. At 10.16 GHz, the surface current is strong at the boundaries of the innermost ring, and lower and upper half current flows in the opposite directions, as shown in Fig. 7b. This surface current induces magnetic fields at the periphery of the innermost ring, as depicted in Fig. 8b. A strong electric field exists adjacent to the horizontal sides and gap coupling stips of the innermost ring (shown in Fig. 9b), indicating the contribution of the innermost ring for the resonance at 10.16 GHz.

### Array analysis and measurement

**Figure 10:**
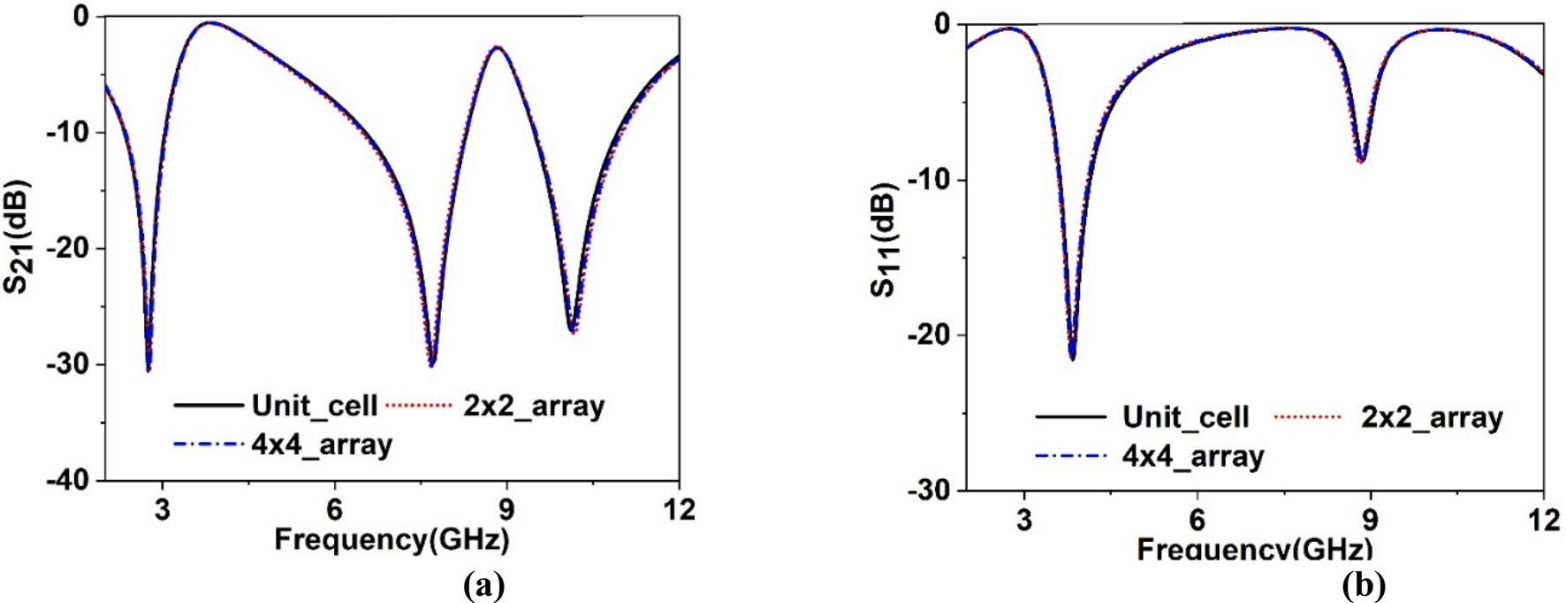
Comparison of array response with unit cell: (**a**) S_21_, (**b**) S_11_.

**Figure 11:**
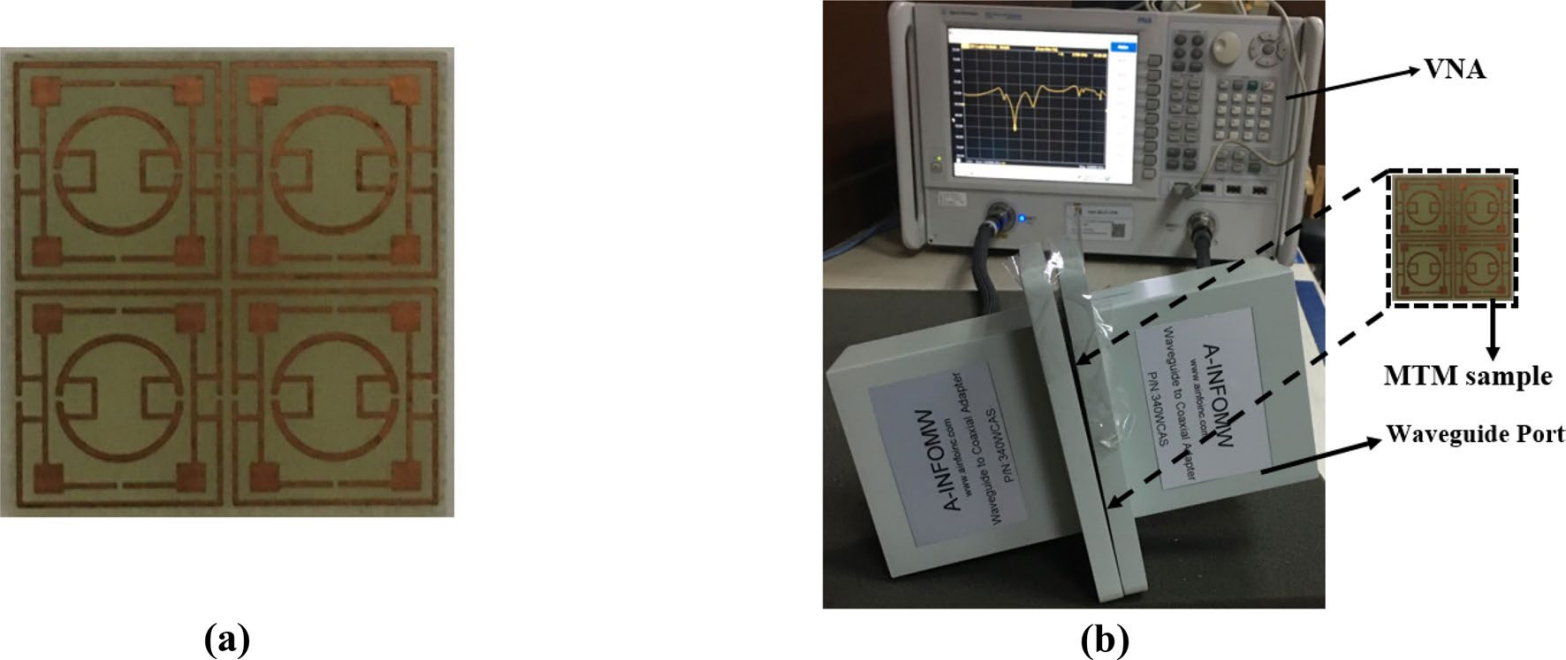
(**a**) Prototype of 2 × 2 array of proposed MTM, (**b**) measurement setup using VNA and waveguide port.

**Figure 12:**
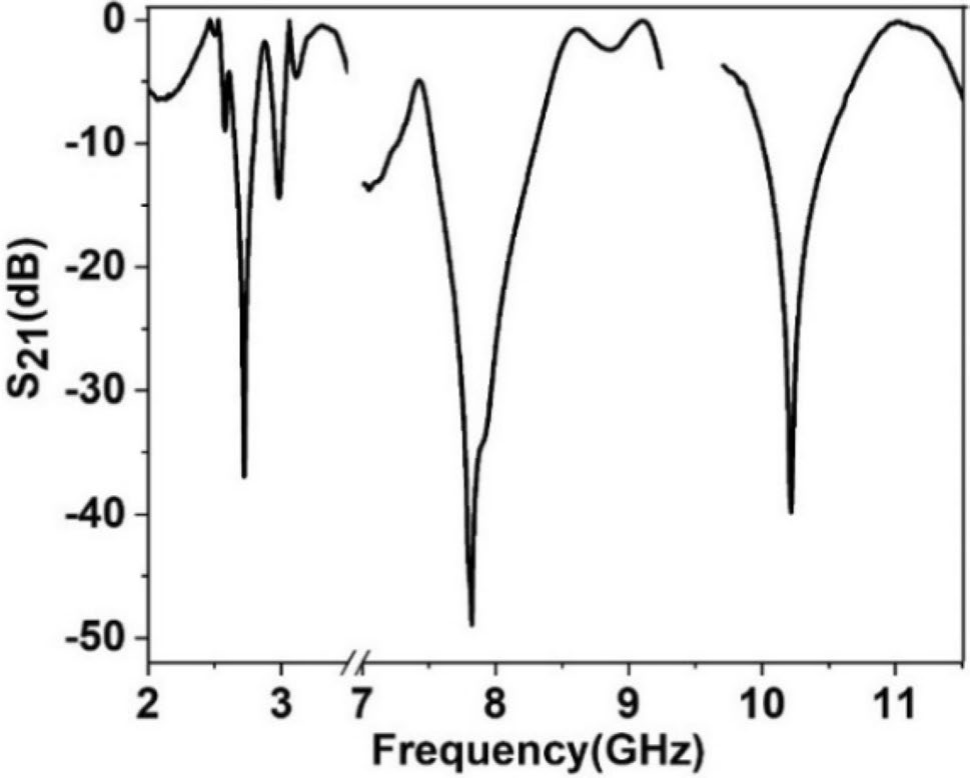
Measured S_21_ of the proposed MTM.

As in many pragmatic cases, the array of the unit cells works together for particular applications rather than solitary unit cell and so, outcomes of array is analyzed using 2 × 2 and 4 × 4 arrays of MTM cells. The S_21_ and S_11_ obtained from this study are depicted in Fig. 10a,b, respectively. Comparing the array performances with the unit cell indicates that array results are well matched with the unit cell. The substantial similarity is owing to the fact that the MTM cell is symmetric in the structure; thus, when several cells are organized in a regular pattern in an array, the electromagnetic field near the adjacent side does not affect much. Therefore, it avoids harmonics and resonance frequency shifts. Since unit cell response and array response of the metamaterial are similar, so measurement result is taken by fabricating a 2 × 2 array. The fabricated prototype is shown in Fig. 11a, whereas measurement arrangement is presented in Fig. 11b. In the measurement setup in Fig. 11b, two waveguide ports are employed as a transmitter and receiver of the electromagnetic waves that are coupled to the vector network analyzer (VNA). MTM array is positioned between two ports. As displayed in Fig. 12, the measured result shows three significant resonances of S_21_ at 2.72 GHz, 7.82 GHz and 10.21 GHz with a magnitude of − 36.9 dB, − 48.9 dB, − 39.8 dB, respectively. It is also observed from this figure that some amount of noise and harmonics exist at the low and mid-frequency in the measured S_21_. Moreover, measured S_21_ deviates from simulation one in terms of resonance frequencies and magnitude at resonance, but this mismatching is not significant. Fabrication tolerances, coupling effect in the waveguide ports and calibration errors in VNA are the reasons for this mismatching. Despite minor mismatch and harmonics, the measured result is well agreed with the simulation results covering the S, C and X-bands.

### Comparative analysis

A comparison is made of the proposed MTM unit cell with some recent works where MTMs are designed to target various applications, summarized in Table 3. In this comparison, physical and electrical dimensions of the applied MTM, Transmission coefficient resonance frequencies, effective medium ratio (EMR), covering bands are considered major parameters. Moreover, special features along with the applications are also discussed in this comparison. EMR is calculated at the lowest resonance frequency of S_21_ using the relation, $\mathrm{EMR }=\uplambda /L$, where $\uplambda$ is the wavelength at the lowest resonance frequency of S_21_ and *L* is the highest dimension of the MTM unit cell. From Table 3, it is observed that in Refs.^1,28,41–43^, the sizes of the listed MTMs are small compared with our proposed MTM, but all these MTMs bear lower values of the EMR compared to our present work. Moreover, Refs.^1,41–43^ covers only a single band, limiting their applications to a specific range of frequencies. Although MTM in Ref.^28^ covers X, C and Ku bands, no particular application is presented. All the other MTMs presented in Refs.^44–47^ in Table 3 lag behind physical size, EMR values, and the number of covering bands. Thus, the proposed MTM covers multiples bands with high EMR values within its moderate dimension that provides the flexibility of application in small dimension devices in wireless communication with a diversity of operating in the wider frequency range. Moreover, its negative permittivity property with near zero refractive index and permeability makes it suitable for gain enhancement verified with numerical analysis in the next section.

**Table 3:** Comparison of the various parameters of the proposed antenna with some other recent works on MTM.

References	Year	Dimensions: physical (mm) and electrical (λ × λ)	Resonance frequency(GHz)	EMR	Covering bands	Features with application
^1^	2020	8 × 8 0.07λ × 0.07λ	3.5	10.71	S	1. Coupling reduction in MIMO antenna system 2. Meander line-based diagonally coupled MTM
^17^	2021	8 × 8 0.063λ × 0.063λ	2.38, 4.24, 5.98, 9.55, 12.1, 14.34	15.75	S, C, X, Ku	1. Symmetric about the vertical axis 2. Frequency tuning is accomplished through shunt inductance 3. Frequency shifting occurs in arrays due to mutual coupling between the array elements 4. Applied for antenna gain enhancement. At high-frequency, the gain increment is not significant
^28^	2020	9 × 9 0.125λ × 0.125λ	4.15, 10.84, 14.93	8.03	C, X, Ku	1. Radar and satellite application (proposed) 2. Coupled split ring resonator with cross-shaped interconnection in innermost rings 3. Negative permittivity
^41^	2020	6 × 5 0.8λ × 0.67λ	40	1.25	Ka	1. Gain increasing and mutual coupling reduction 2. Modified peace logo planar-based metamaterial 3. MTM slabs are operated as the lens
^42^	2021	4 × 4 0.11λ × 0.11λ	8.5	8.8	X	1. Gain enhancement and SAR reduction 2. Polygonal split rings are used as MTM 3. Negative permeability
^43^	2021	10.3 × 10.3 0.59λ × 0.59λ	17.1	1.7	Ku	1. Application in LTE 46/WLAN and Ka-band antenna for gain improvement 2. Asymmetric truncated circular metal patch with copper back 3. Single negative MTM
^44^	2019	15.6 × 15.6 0.14λ × 0.14λ	2.65, 4	7.25	C, S	1. Mutual coupling suppression in antenna 2. Symmetrical SRR with double slot and inductive stub at inner ring 3. Epsilon negative MTM
^45^	2017	25 × 25 0.23λ × 0.23λ	2.8	4.28	S	1. Sub-GHz microwave applications (proposed) 2. Flexible SRR based MTM loaded with ferrite at the back
^46^	2018	20 × 20 0.77λ × 0.77λ	11.5,13.5	1.4	X, Ku	1. Applications in pressure sensing 2. MTM consists of mirror reflexed C loaded resonator inside the rings with the copper ground
^47^	2016	35 × 35 0.21 λ × 0.21λ	1.75	4.9	L	1. S-shaped resonator circled with ground frame and FTL structure 2. Applicable in sensing
Proposed	2021	10 × 10.5 0.09λ × 0.097λ	2.78, 7.7, 10.16	10.27	S, C, X	1. Gain and directionality enhancement of broadband monopole antenna 2. Symmetrical MTM consists SRRs with coupling near the split gaps 3. Negative permittivity, with near zero permeability and refractive index

## Design and simulation of the test antenna

**Figure 13:**
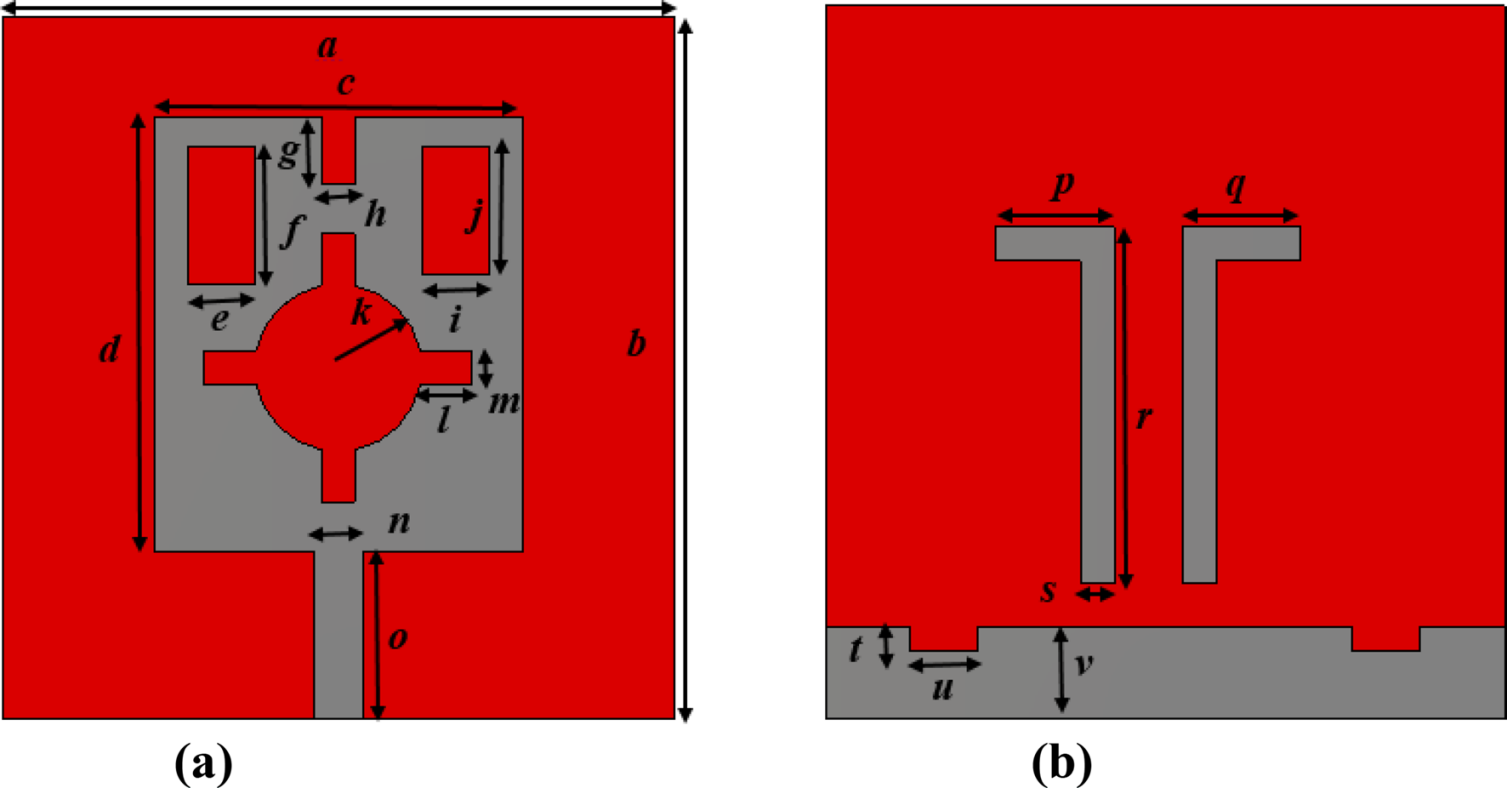
(**a**) Front view of the antenna (**b**) back view (CST STUDIO SUITE 2019, https://www.3ds.com/products-services/simulia/products/cst-studio-suite)^38^.

**Figure 14:**
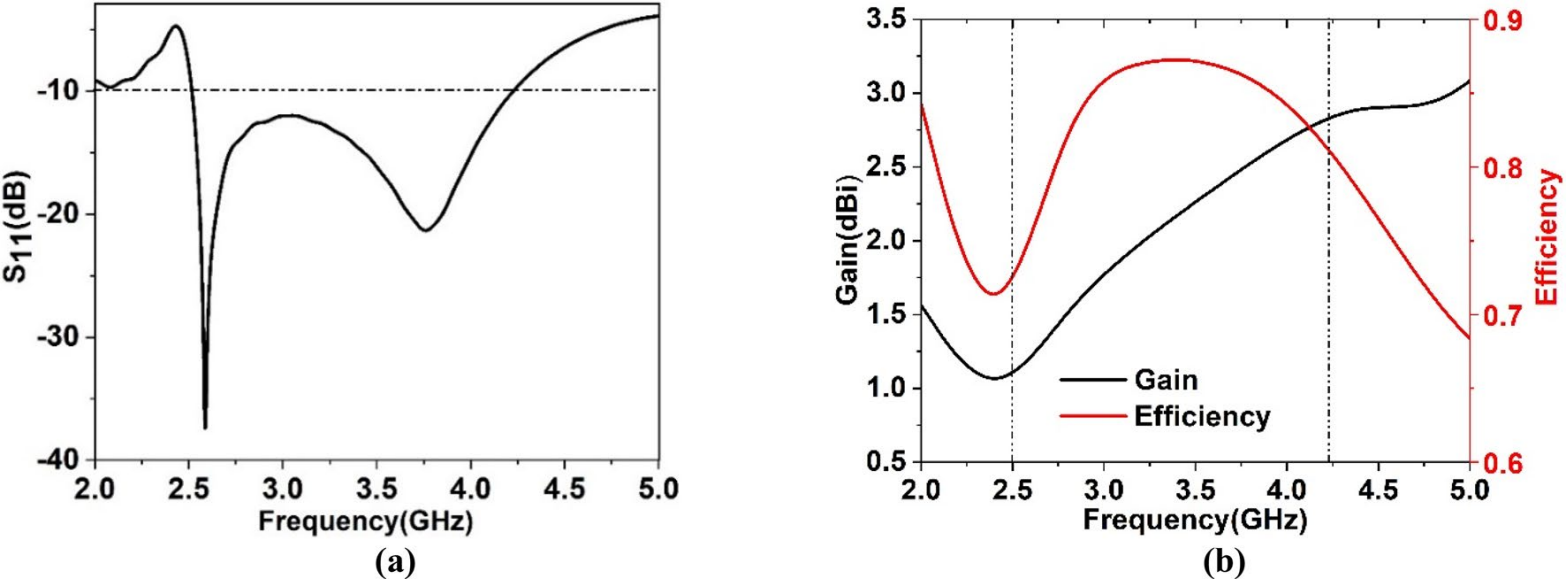
(**a**) reflection coefficient (S_11_) plot of the proposed antenna (**b**) gain and efficiency plot.

**Figure 15:**
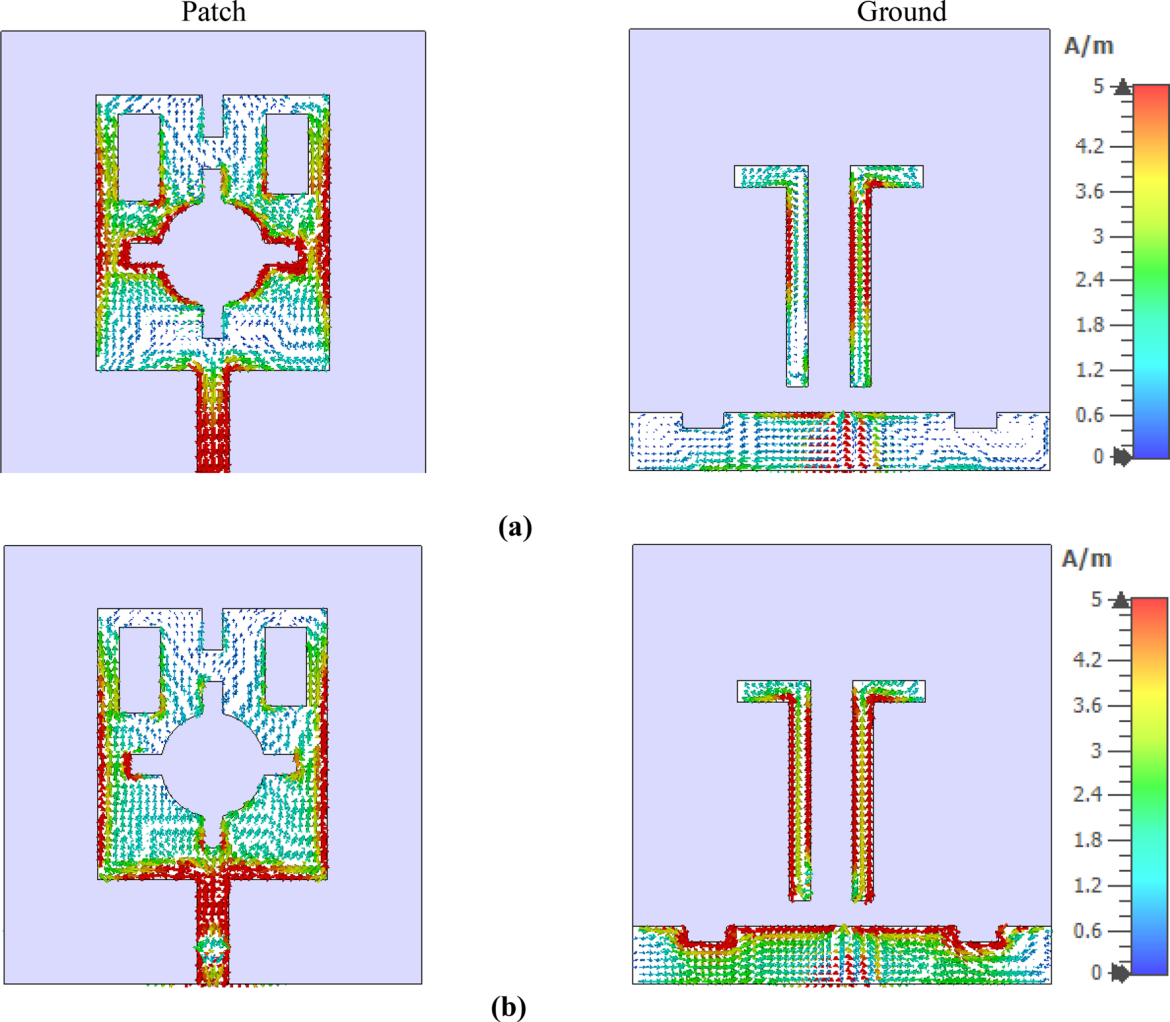
Surface current distributions at patch and ground side at (**a**) 2.60 GHz and (**b**) 3.77 GHz (CST STUDIO SUITE 2019, https://www.3ds.com/products-services/simulia/products/cst-studio-suite)^38^.

A monopole antenna is designed consisting of a slotted patch with partial ground and parasitic elements in the backside, as shown in Fig. 13. The designed antenna is initiated on an FR4-substrate with 1.6 mm thickness and 4.3 dielectric constant. The overall dimension of the antenna is 40 × 41 mm^2^ and has a patch of 22 × 26 mm^2^. At the same time, a feedline of width 2.96 mm is used to match the impedance to 50 Ω. Various slots in the patch are used to control the current flowing through the patch to obtain the wideband response with sufficient gain and efficiency of the antenna^48^. Figure 13a shows the geometry of the patch of the antenna, whereas Fig. 13b shows the structural geometry of the ground plane. The different parameter values of the various slots and segments in patch and grounds are listed in Table 4. The partial ground structure helps to obtain an omnidirectional radiation pattern, whereas slots in the ground plane along with the inverted L shaped parasitic elements help to improve the bandwidth. Figure 14a shows the reflection coefficient of the antenna that expresses the bandwidth extended from 2.5 to 4.24 GHz, with two resonances occurring at 2.6 GHz and 3.77 GHz. Moreover, the efficiency and gain presented in Fig. 14b indicates that the average efficiency is more than 80% within this band, and the antenna's average gain is 2 dBi with maximum efficiency of 88% and a maximum gain of 2.95 dBi. The slots inside the patch modify the direction of the current flow and lessen the patch's dimension^49^ or help to improve the impedance bandwidth^50^. The effects of various slots on resonance phenomena surface current analysis can be done for two different frequencies of resonances at 2.6 GHz and 3.77 GHz. Figure 15 shows the current distribution at the patch and ground side of the antenna. As expressed in Fig. 15a, it is noticed that current is more concentrated near the vertical edges of the patch and edges of the inner slots. Larger current path due to inner slot helps to increase the effective inductive reactance. Thus, it causes to obtain the resonance frequency at 2.6 GHz. On the other hand, a study of ground side current (in Fig. 15a) for this frequency of resonance shows that parasitic elements have a high concentration of currents. In contrast, the ground plane offers a lower dense current. Unlikely, as shown in Fig. 15b, at 3.77 GHz, patch current is mainly concentrated at the vertical edges of the patch, whereas surrounding the inner slot, the current is drastically reduced compared to the current of earlier resonance. Due to the reduced current path, the inductance associated with the patch is lessened, which ultimately affects the resonance at this frequency. A closer look at the ground plane current (in Fig. 15b) shows that current density at both parasitic elements is significantly higher compared with the current through the same elements at 2.6 GHz. A high-intensity current is noticed in the ground plane at the top horizontal edges and through slots in the ground plane. Moreover, all over the ground plane, the current density is high as compared to the current flowing through the ground at 2.6 GHz. Thus, it can be concluded that the current surrounding the inner slot of the patch contributes significantly to the resonance at 2.6 GHz. In contrast, resonance at 3.77 GHz is mainly governed by the current flowing through the backplane slots.

**Table 4:** Parameter values of the antenna.

Parameter	Dimension (mm)	Parameter	Dimension (mm)	Parameter	Dimension (mm)	Parmeter	Dimension (mm)	Parmeter	Dimension (mm)
a	40	b	41	c	22	d	26	e	4
f	8.2	g	4	h	2	i	4	j	7.6
k	5	l	3	m	2	n	2.96	o	10
p	7	q	7	r	21	s	2	t	1.5
u	4	v	5.5

## Gain enhancement of antenna using MTM superstrate with antenna

**Figure 16:**
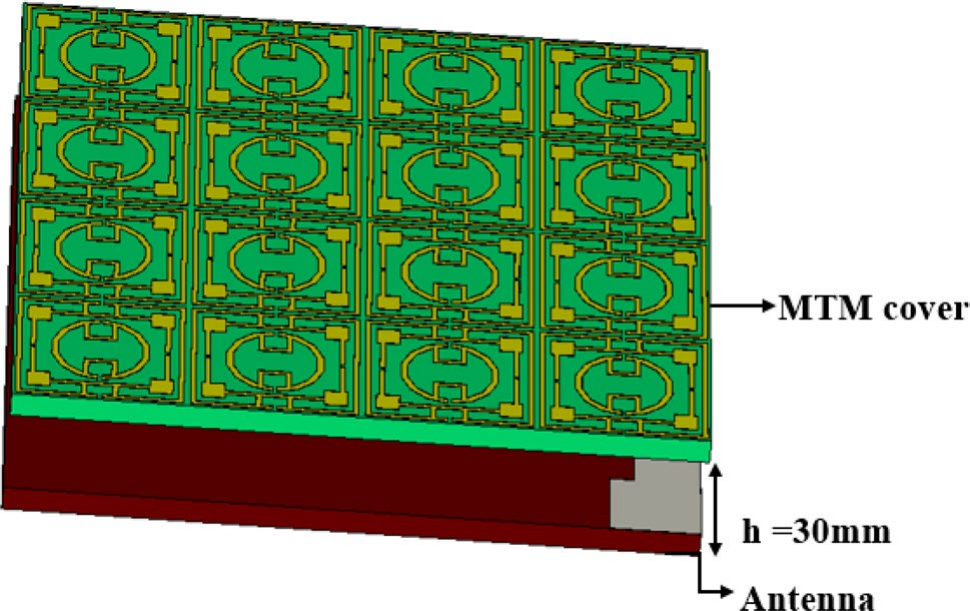
Antenna with MTM superstrate (CST STUDIO SUITE 2019, https://www.3ds.com/products-services/simulia/products/cst-studio-suite)^38^.

**Figure 17:**
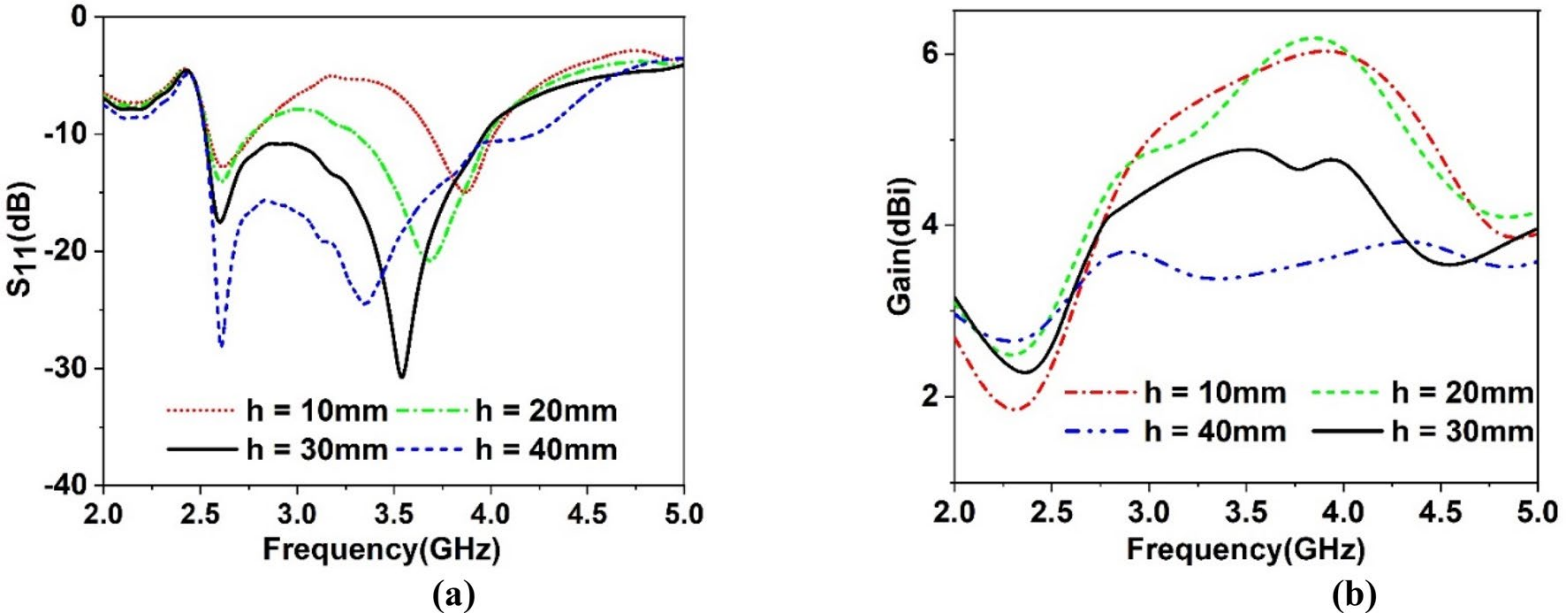
Antenna performance for different distance, h between antenna and MTM superstrate: (**a**) S_11_ and (**b**) gain.

**Figure 18:**
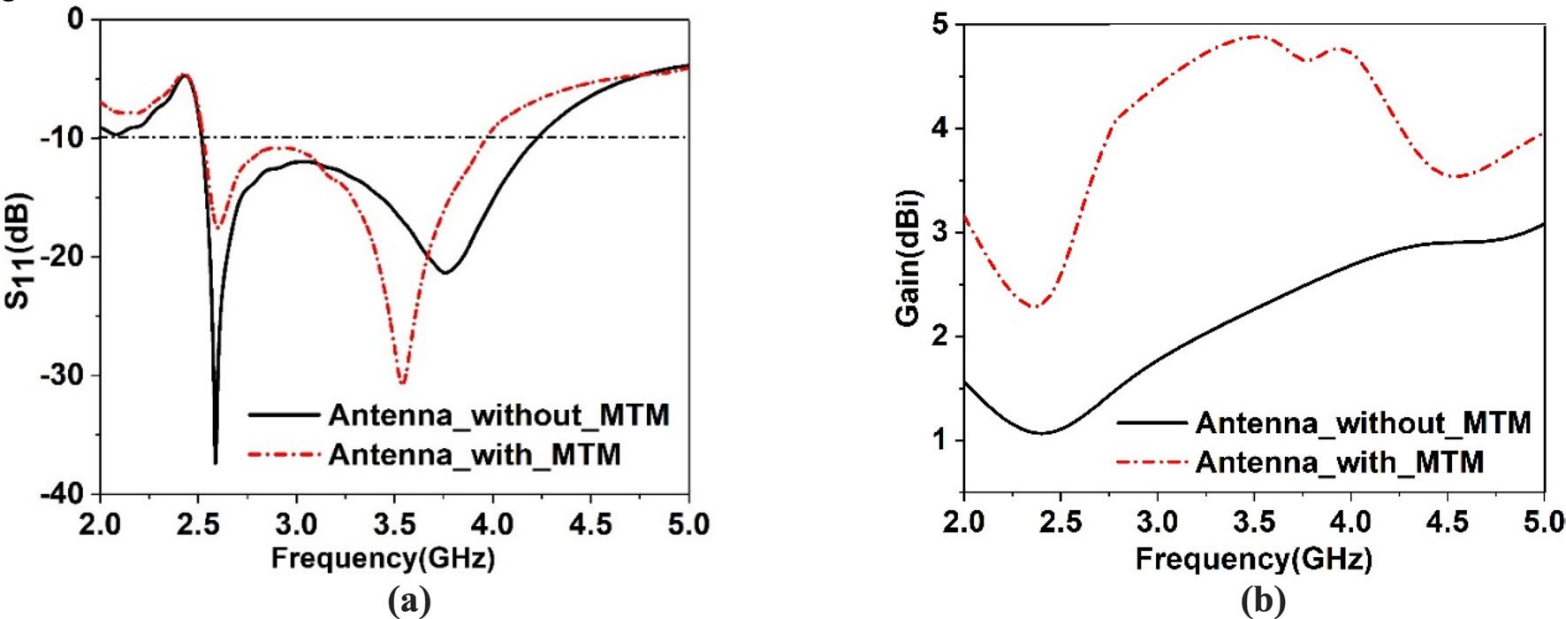
Comparison of antenna outcomes with and without MTM: (**a**) reflection coefficient (S_11_), (**b**) gain.

A metamaterial array is introduced, having a 4 × 4 array with the overall dimension of 41 mm × 40 mm to use it as a superstrate. This array element provides the same area of the designed antenna that covers the total surface of the antenna. Thus, it interacts with the most emitted radiation of the antenna, which ultimately helps to improve the gain of the antenna. Figure 16 shows the MTM with the antenna in which MTM array is placed as superstrate at the ground side of the antenna at spacing of 30 mm. The space is selected by parametric study targeting that position of MTM array will not affect the bandwidth of the antenna much rather it will increase the gain. Figure 17 shows the parametric study for various distances between antenna and MTM cover. The S_11_ response of the antenna showed in Fig. 17a for different distances between antenna and MTM. As shown in Fig. 17a, the antenna shows dual-band resonances when h = 10 mm for the low distance. As the value of h increases, lower band resonance frequency seems to maintain a constant value with increasing magnitude with distance. But in the case of the upper band, magnitude and resonance frequency shift with increasing the distance. As the distance increases, the upper band resonance frequency decreases gradually with the increasing distance. Eventually, two different bands come closer and combine, and a wideband response is perceived when h = 30 mm or higher. A comparison of antenna gain with MTM for different values of h is made, and the gain plots are depicted in Fig. 17b. As expressed in Fig. 17b, high gain is observed when the distance between the antenna and MTM is low and decreases gradually with the increasing distance. It is because when the distance between the antenna and MTM is low, most of the radiated field impinges on the MTM. Thus more directional radiation is obtained due to the near zero property of the MTM. As the distance increases, more radiated field propagates through the free space and thus, MTM faces less radiation to make its direction, resulting in a decreased gain. Therefore, from this parametric analysis, an optimum distance of 30 mm is considered that provides a considerable bandwidth extended from 2.5 to 3.96 GHz with a moderate amount of enhanced gain. A comparison of S_11_ of the antenna with and without MTM is presented in Fig. 18a. It is observed that bandwidth is decreased for the antenna with MTM with a downward shift of upper cutoff frequency. It can be explained by using the impedance matching of the equivalent circuit of the antenna as impedance, Z and frequency, *f* of resonance can be expressed^51^ as, $Z=\frac{1}{j\omega C+1/1/j\omega L}$ and $f=\frac{1}{2\pi \sqrt{L{C}}}$ respectively, in which L corresponds to total inductance and C is the capacitance. Introducing the MTM with antenna causes a modification of the total capacitance, thus resonance frequency shifts. As the distance between antenna and MTM is low, MTM causes the reduction of the total capacitance of the antenna equivalent circuit, which eventually causes the higher resonance frequency, as shown in Fig. 17a. Contrarily, an increasing distance effects as changing resonances towards lower frequencies.

**Figure 19:**
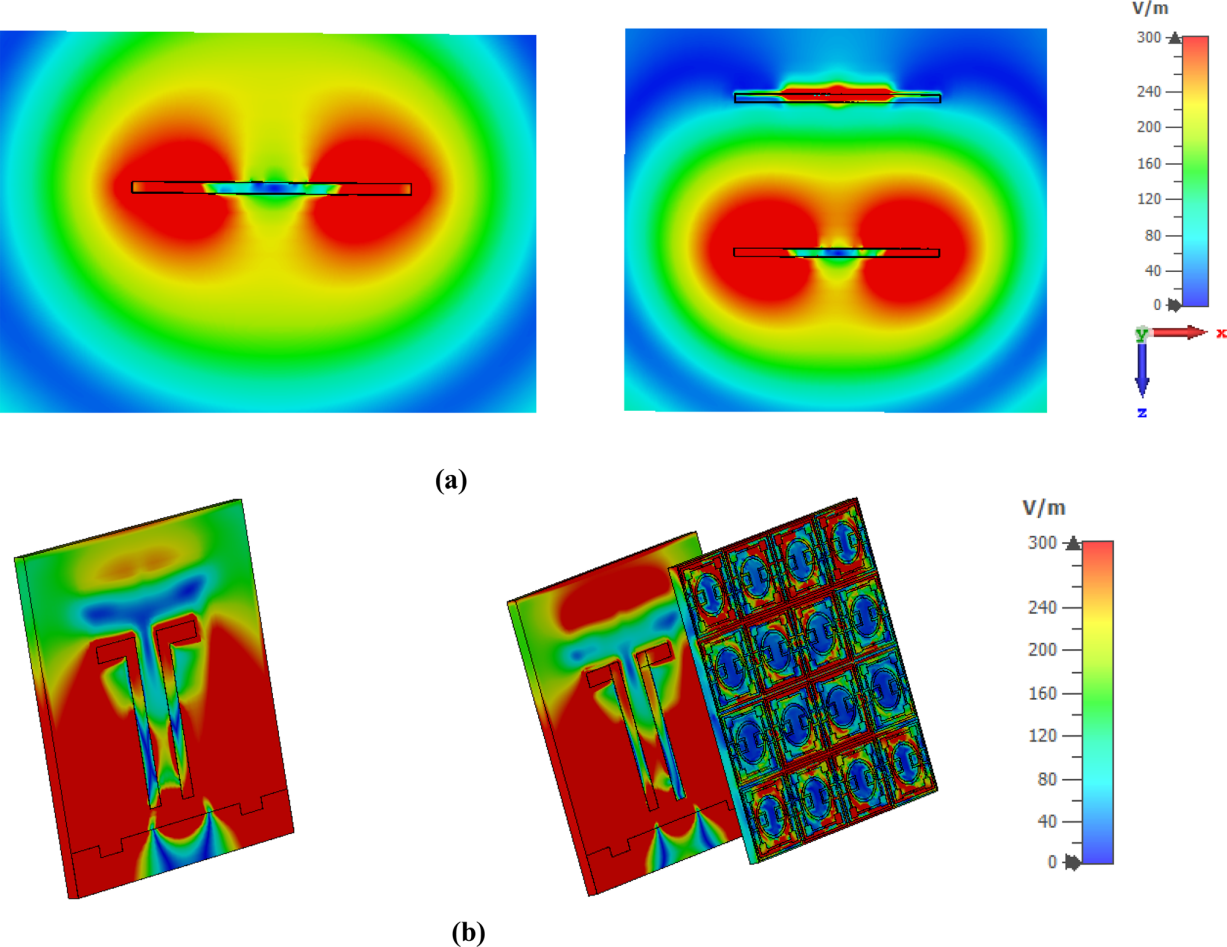
Electric field distribution at 3.77 GHz with and without MTM: (**a**) at XZ plane surrounding antenna, and (**b**) on the surface of antenna and MTM superstarte (CST STUDIO SUITE 2019, https://www.3ds.com/products-services/simulia/products/cst-studio-suite)^38^.

**Figure 20:**
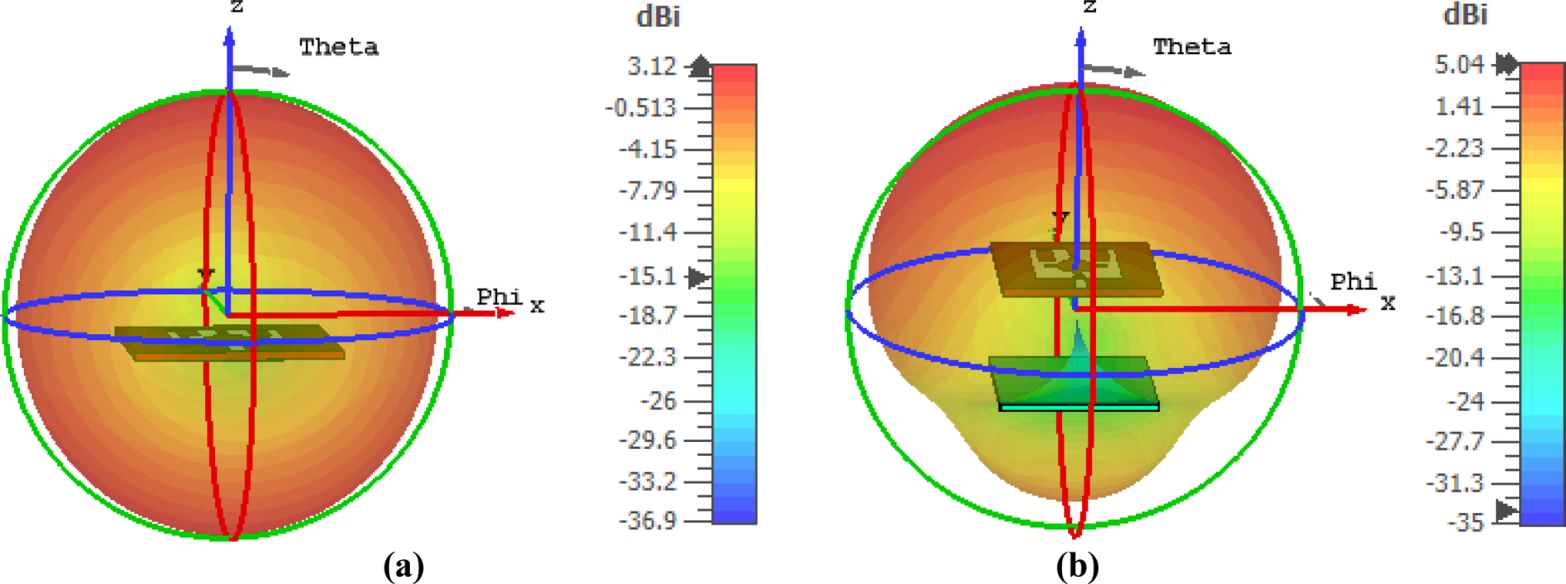
3D radiation pattern at 3.77 GHz for: (**a**) antenna without MTM and (**b**) antenna with MTM superstarte (CST STUDIO SUITE 2019, https://www.3ds.com/products-services/simulia/products/cst-studio-suite)^38^.

A comparison of the gain for the antenna with and without MTM superstrate is shown in Fig. 18b that indicates a high gain with MTM superstrate. MTM cover provides a maximum gain of around 4.95 dBi, whereas bared antenna offers a maximum gain of around 2.5 dBi, indicating a gain enhancement by 95%. The gain enhancement can be explained with the help of the electric field (E) distribution in space and on the antenna. This distribution of the E fields at 3.77 GHz is presented in Fig. 19a,b, respectively. Figure 19a shows that electric field strength increases significantly surrounding the antenna when metamaterial is introduced. Moreover, when a radiated field is exposed over MTM, it emits radiation due to its NZI property which is normal to the surface of the MTM. Additionally, EM waves that incidents on the MTM contain a sideward E component that becomes more directional due to the NZI property, which added an extra field in a particular direction. Thus, antenna gain increases significantly. A comparison of the E field distribution over the antenna is shown in Fig. 19b. This figure shows a strong E field induced at the top of the antenna when MTM is used with the antenna. This strong E field is also the contributor to the gain enhancement. Thus, MTM superstrate causes to increase the directionality as well as radiated power that combinedly enhances the overall gain of the antenna. Figure 20a shows the 3D radiation pattern at 3.77 GHz for the antenna without MTM that is omnidirectional in nature. When MTM superstrate is used, the radiation pattern becomes directional as shown in Fig. 20b. The radiation is more concentrated in the Z direction, increasing the antenna's directional gain when MTM superstrate is used.

**Figure 21:**
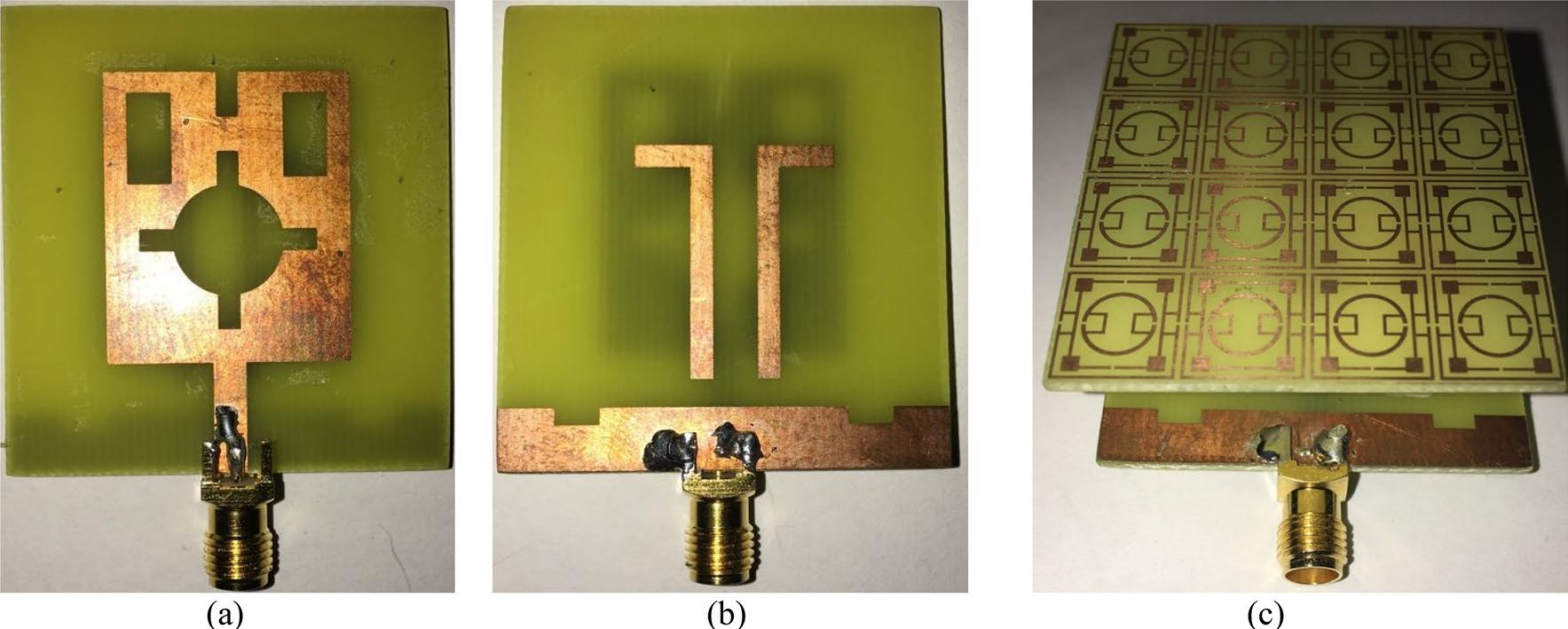
Fabricated prototype of antenna; (**a**) front view (**b**) back view (**c**) antenna with MTM array.

**Figure 22:**
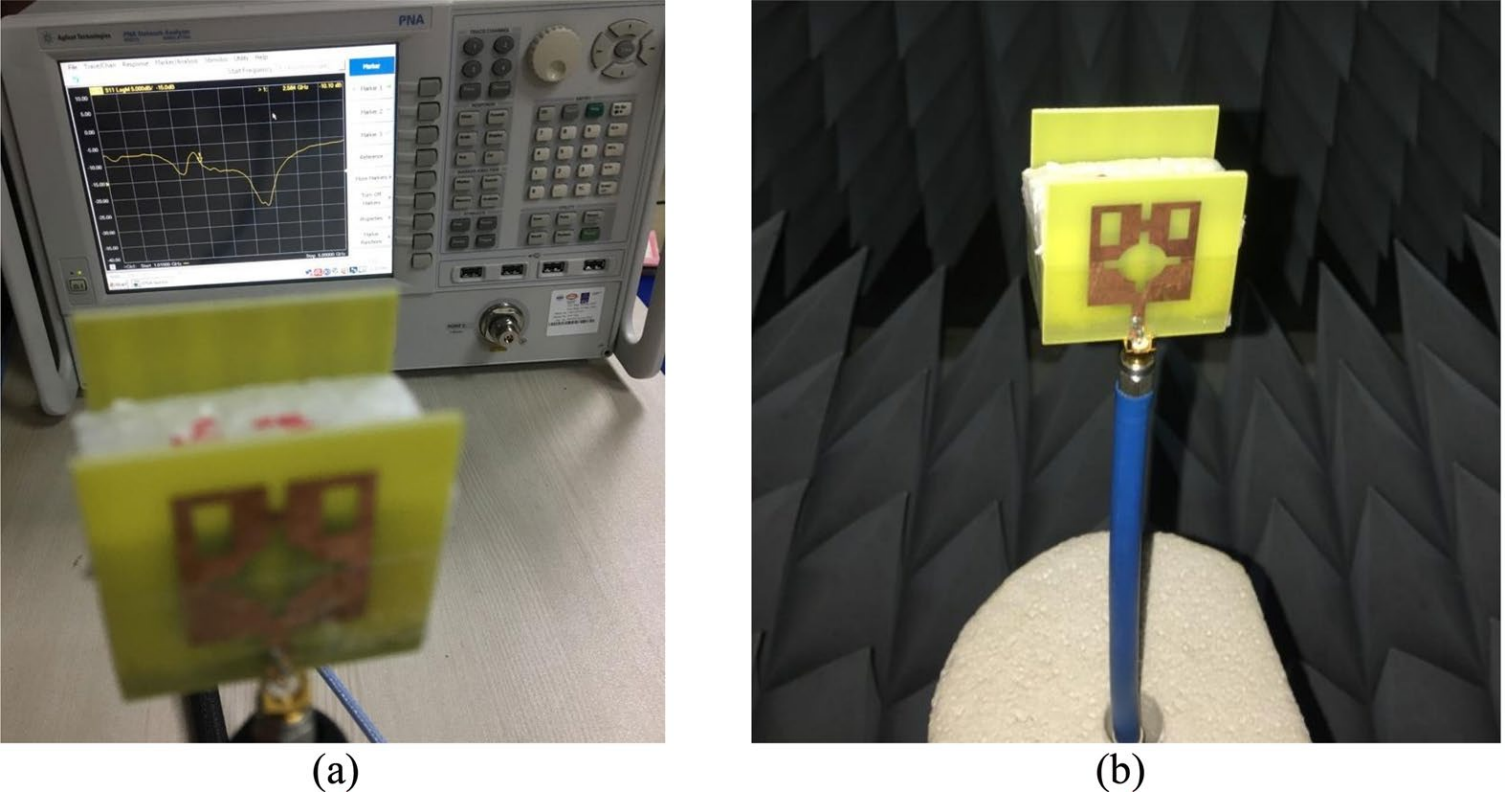
Experimental setup for the antenna with MTM: (**a**) S_11_ measurement using Vector Network Analyzer (VNA), (**b**) measurement arrangement using Satimo Nearfield measurement system.

**Figure 23:**
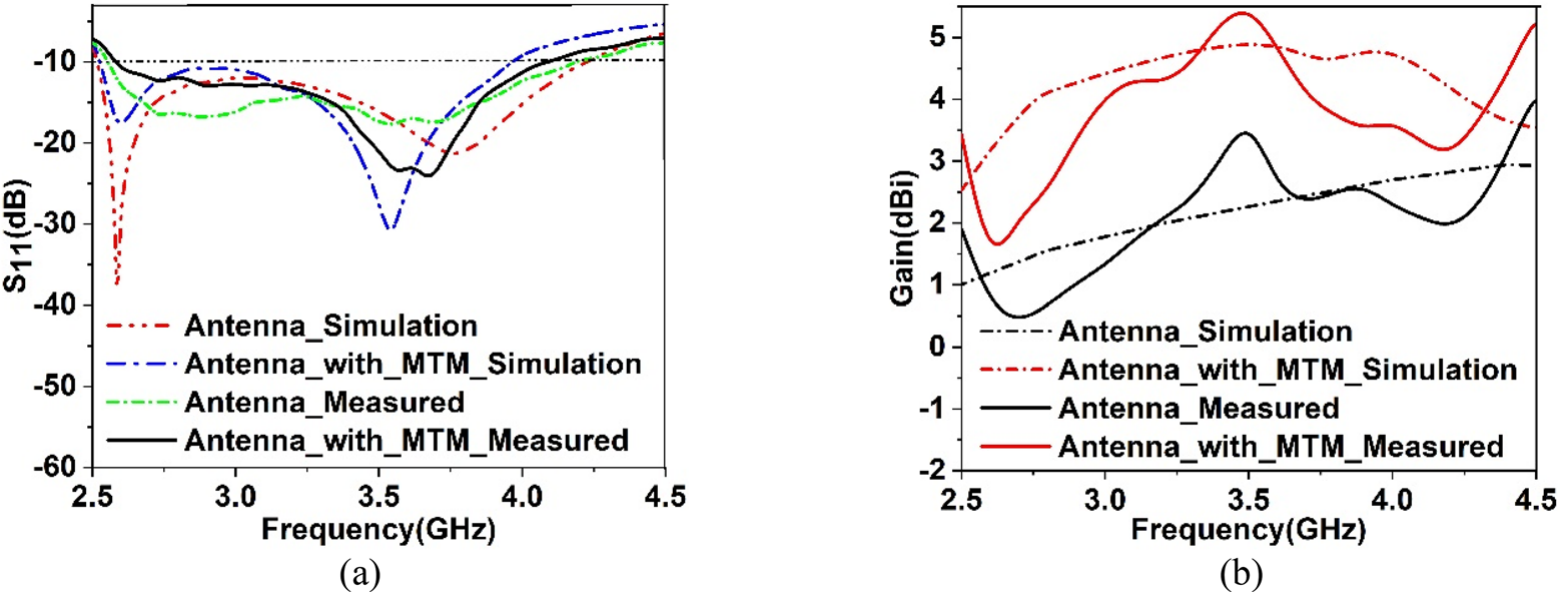
Plots of (**a**) measured and simulated S_11_, (**b**) measured and simulated gain.

**Figure 24:**
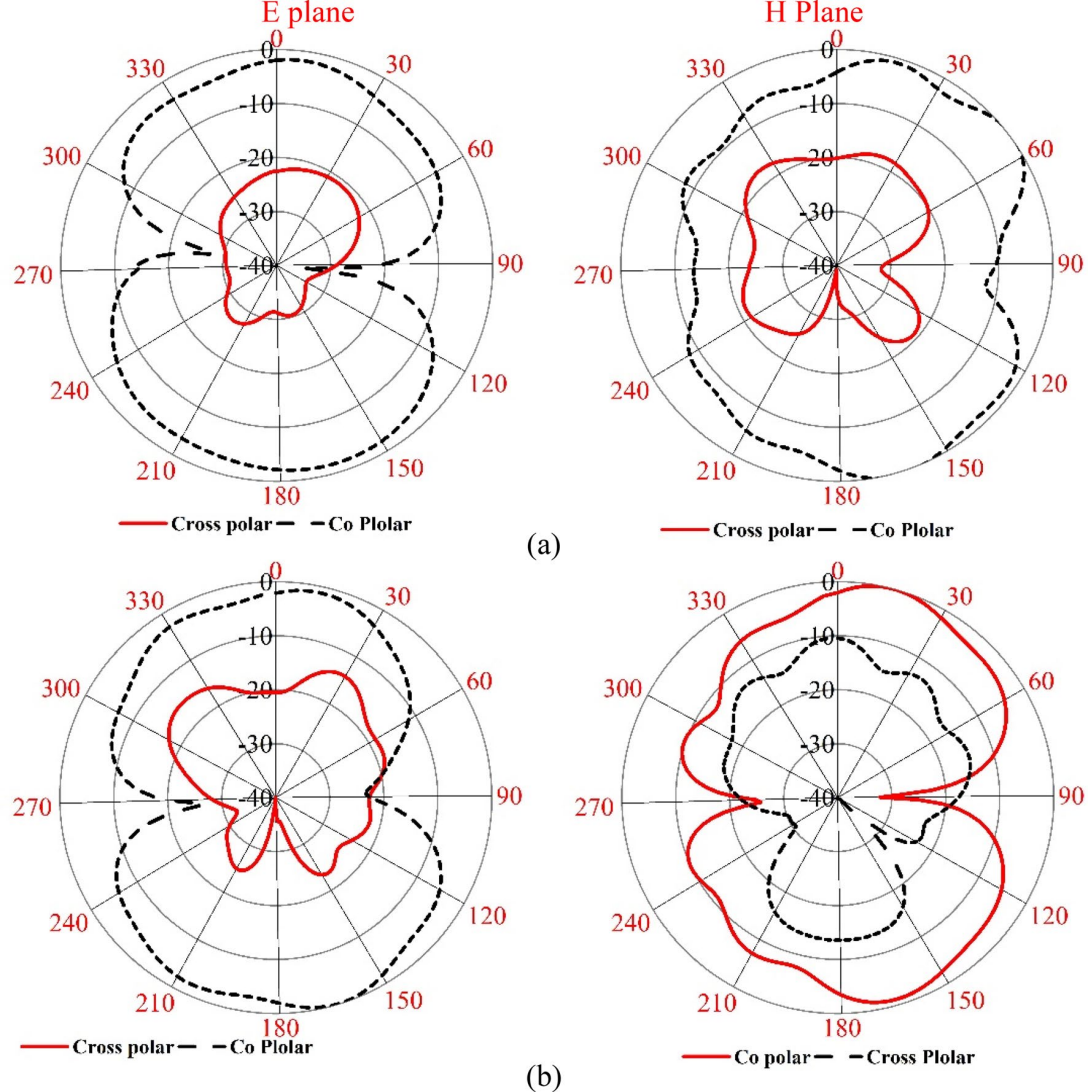
2D measured radiation pattern of the antenna only at (**a**) 2.60 GHz and (**b**) 3.76 GHz.

**Figure 25:**
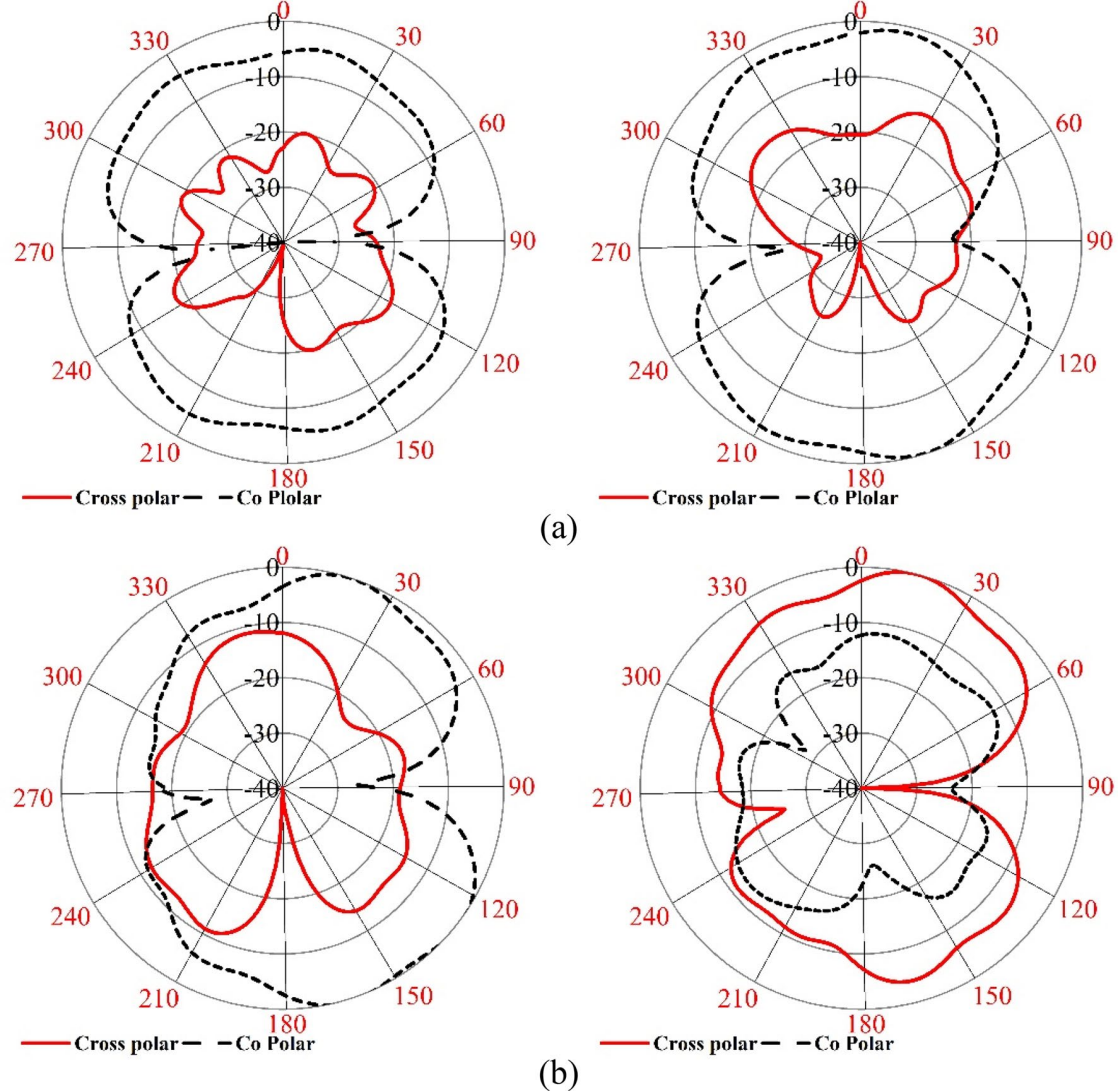
2D measured radiation pattern of the antenna with metamaterial at (**a**) 2.60 GHz and (**b**) 3.76 GHz.

The result obtained in the simulation is verified by fabricating the antenna and taking the measurement for S_11_ and gain. Figure 21a shows the front view of the antenna prototype, whereas Fig. 21b exhibits the back view. Morevoer, antenna with metamaterial superstrate arrangement is presented in Fig. 21c where 4 × 4 array of unit cells of the proposed MTM is seperated from the antenna using a 30 mm thick polystyrene block. The experimental arrangement for S_11_ is depicted in Fig. 22a, in which a vector network analyzer (VNA) is used for measuring this parameter. The measured values of the S_11_ for antenna with and without metamaterial is depicted in Fig. 23a in comparison with outcomes of simulation. From Fig. 23a, it is noticed that measured results of the antennas with and without metamaterail exhibits close similarity. The measured − 10 dB impedance bandwidth of the test antenna without MTM is exteded from 2.56 to 4.2 GHz with a resonance peak at 3.5 GHz having magnitude of − 17 dB. When MTM is used as the superstrate − 10 dB bandwidth is observed extending from 2.58 to 4.1 GHz with resonance peak of – 24 dB at 3.67 GHz. In both cases, the bandwidth is slightly less than the simulation result, but the deviations are less than 5%. This slight variation is associated with fabrication tolerances, calibration errors involved in VNA, and loss incurred in the cable connecting VNA and the antenna prototype. But neglecting these errors fabricated antenna provides good bandwidth when metamaterial superstrate is used. Figure 22b shows the Satimo nearfield measurement setup for the antenna with MTM superstrate to determine the performance parameters, including gain and radiation pattern. Corresponding gain data is plotted in Fig. 23b to compare measured gain with an antenna without MTM and simulation results. This result shows that measured antenna gain with and without metamaterial is slightly varied compared to the simulation result. This mismatching is due to fabrication tolerances of the antenna and calibration errors associated with the Satimo near field measurement system. Measured antenna gain is less compared compared to the simulated gain except the frequency range 3.2–3.8 GHz where the measured gain well above the simulated gain. But, comparing measured gain between antenna without metamaterail and antenna with metamaterial it is observed that metamaterial exhibits its dominant impact on the antenna and helps to boost up the antenna gain. Figures 24 and 25 display the measured 2D radiation pattern of the designed antenna and antenna with metamaterial array for E and H plane at 2.6 GHz and 3.76 GHz respectively. From Fig. 24, it can be stated that the radiation pattern shows an omnidirectional type for the antenna with low cross-polarization. On the otherhand, for antenna with metamaterial presented in Fig. 25, the pattren shows slightly directional towards + z directions with low cross polarizations due to gain enhancement by using metamaterial array.

The performance of the proposed antenna with MTM superstrate is compared with some other recently published MTM loaded antennas, and the comparison outcomes are presented in Table 5. From Table 5, it is observed that the antenna with MTM superstrate presented in Ref.^52^ provides a good maximum gain of 6.56 dBi with an operating resonance frequency at 2.4 GHz. But the overall dimension of the antenna system is large compared to our proposed antenna system. Moreover, the bandwidth of this antenna system is low compared, and metamaterial helps to increase the gain by 22.37%. As is viewed from this Table 5, Ref.^53^ shows a comparatively high gain with good percentage enhancement due to the MTM superstrate. But the overall dimension of the MTM array and antenna is high, and this gain is achieved by using four layers of MTM superstrates. Moreover, this antenna operates at a high frequency with a comparatively low bandwidth. In Ref.^54^, the triangular-shaped antenna array is used for two ports MIMO system that exhibits the highest gain of 14.05 dBi. But, this high gain is due to an antenna structure of large size. In this antenna system, a comparatively low gain enhancement is acquired when MTM array is used as a superstrate. Contrary to this, the dimension of the antenna system presented in Ref.^55^ exhibits lower dimension, moderate antenna gain, with good gain enhancement due to MTM superstrate. But the high gain within the lower dimension is achieved by using two layers of superstrate. Thus, comparing these antenna systems with MTM superstrate, our proposed MTM provides good gain enhancement within its small dimension with a broader bandwidth. Thus, the proposed MTM can be used for developing compact-sized high gain broadband antenna system. The antenna presented in this article exhibits − 10 dB S_11_ response from 2.58 to 4.1 GHz having a bandwidth of 1.52 GHz. With MTM superstrate, it exhibits maximum gain of 5.5 dBi. This broadband compact high gain antenna can be used for broadband wireless applications like microwave based energy harvesting from WiMAX, WLAN or from sub-6 GHz 5G sources.

**Table 5:** Comparison of proposed MTM loaded antenna with some other existing MTM loaded antenna.

Ref	Dimension (mm)	Superstrate layer	Operating frequency (GHz)	Bandwidth	Gain (dBi) (maximum)	% Gain enhancement
^52^	61.25 × 61.25	1	2.4	55 MHz	6.56	22.37
^53^	60 × 70	4	9.4	200 MHz	16.1	120.5
^54^	107 × 58	1	6.2	750 MHz	14.05	26.6
^55^	32 × 32	2	5.6	840 MHz	7.26	142
Our work	40 × 42	1	3.76	1.52 GHz	5.5	95

## Conclusion

This paper presents a metamaterial consisting of a resonator patch of a symmetric split ring resonator coupling around the split gaps. This proposed MTM provides three resonances at 2.78 GHz, 7.7 GHz, and 10.16 GHz covering S, C, and X-bands. Due to symmetric structure, mutual coupling between the array elements is reduced, and the array shows a similar S_21_ response of the unit cell. The simulated result is evaluated with the experiment, and the measured result is well matched with the simulation. The equivalent circuit of the MTM is modeled in ADS and validated by comparing the S_21_ response with CST that provides close similarity. The MTM characteristics are also analyzed that show negative permittivity, near zero permeability, and refractive index. The contribution of the different parts of the MTM unit cell in resonance is also studied through the electric field, magnetic field and surface current analysis. The calculated EMR value of 10.7 indicates the compactness of the proposed MTM for application in various small microwave devices. A test antenna having − 10 dB bandwidth of S_11_ extending from 2.5 to 3.95 GHz with maximum gain around 2.5 dBi is designed, and MTM influence is observed by using a 4 × 4 array of MTM as superstrate. MTM superstrate over the test antenna provides a maximum gain of 4.95 dBi with an increment by 95% when the distance between antenna and MTM is 30 mm. NZI property of the proposed ENG metamaterial increases the directionality of the radiation in the XZ plane, which is studied through E field and radiation pattern analysis. The antenna gain enhancement is validated by measuring the performance of the antenna with and without metamaterials. Due to its compactness with high EMR, negative permittivity, near zero permeability and refractive index, proposed MTM can be utilized with various wireless devices in microwave applications, especially to enhance gain and directivity of the antenna.
